# Targeting C3aR/STAT3 signaling attenuates neuroinflammation and reverses neurological impairment in Neuromyelitis optica

**DOI:** 10.1016/j.fmre.2025.11.001

**Published:** 2025-11-07

**Authors:** Li Xu, Xinyi Wang, Huiming Xu, Siqi Chen, Wei Jiang, Haoyang Li, Shabbir Khan Afridi, Yuge Wang, Wenjing Luo, Hui Yang, Changyong Tang, Wei Qiu

**Affiliations:** a Department of Neurology, The Third Affiliated Hospital of Sun Yat-Sen University, Guangzhou 510630, China; b Department of Neurobiology, Heilongjiang Provincial Key Laboratory of Neurobiology, Basic Medical College, Harbin Medical University, Harbin 150081, China; c Department of Medical Retina and Neuro-Ophthalmology, Zhongshan Ophthalmic Center, Sun Yat-Sen University, Guangzhou 510060, China; d Department of Neurology, Shandong Key Laboratory of Mitochondrial Medicine and Rare Diseases, Research Institute of Neuromuscular and Neurodegenerative Diseases, Qilu Hospital of Shandong University, Jinan 250012, China; e Faculty of Biological Sciences, School of Biomedical Sciences, University of Leeds, Leeds LS2 9JT, UK; f Department of Neurology, The First Affiliated Hospital of Guangxi Medical University, Nanning 530021, China

**Keywords:** Neuromyelitis optica (NMO), Microglia, C3aR-STAT3 pathway, Neuroinflammation, Neurological dysfunction

## Abstract

Abundant evidence indicates that the interaction between astrocytes and microglia through the C3–C3a receptor (C3aR) pathway is a crucial contributor to the pathogenesis of neurodegenerative and autoimmune disorders, such as neuromyelitis optica (NMO). However, the role of C3aR in microglia in modulating inflammation related to local lesions and neurological dysfunctions in NMO remains unclear. In this study, we reveal for the first time that the expression of C3aR on microglia is positively correlated with microglial activation, proinflammatory cytokine secretion, and complement deposition, which results in the typical NMO-like lesion characterized by astrocyte injury with aquaporin 4 (AQP4) loss, demyelination, and motor neuron loss, as well as cognitive impairments, visual defects, and locomotor deficits in different models of NMO. The knockdown of C3aR or the use of a C3aR antagonist, both *ex vivo* and in vivo, effectively attenuates neuroinflammation, alleviates pathological lesions, and rescues neurological functions. Mechanistically, we identify STAT3 as a downstream target of microglial C3aR signaling that functionally mediates NMO pathogenesis. Blocking the C3aR/STAT3 cascade restores motor and visual functions by reducing the release of proinflammatory factors. Taken together, our study demonstrates a key role of the C3aR-STAT3 pathway in regulating neuroinflammation in NMO pathology and provides potential targets for therapeutic intervention.

## Graphical abstract

**Figure:**
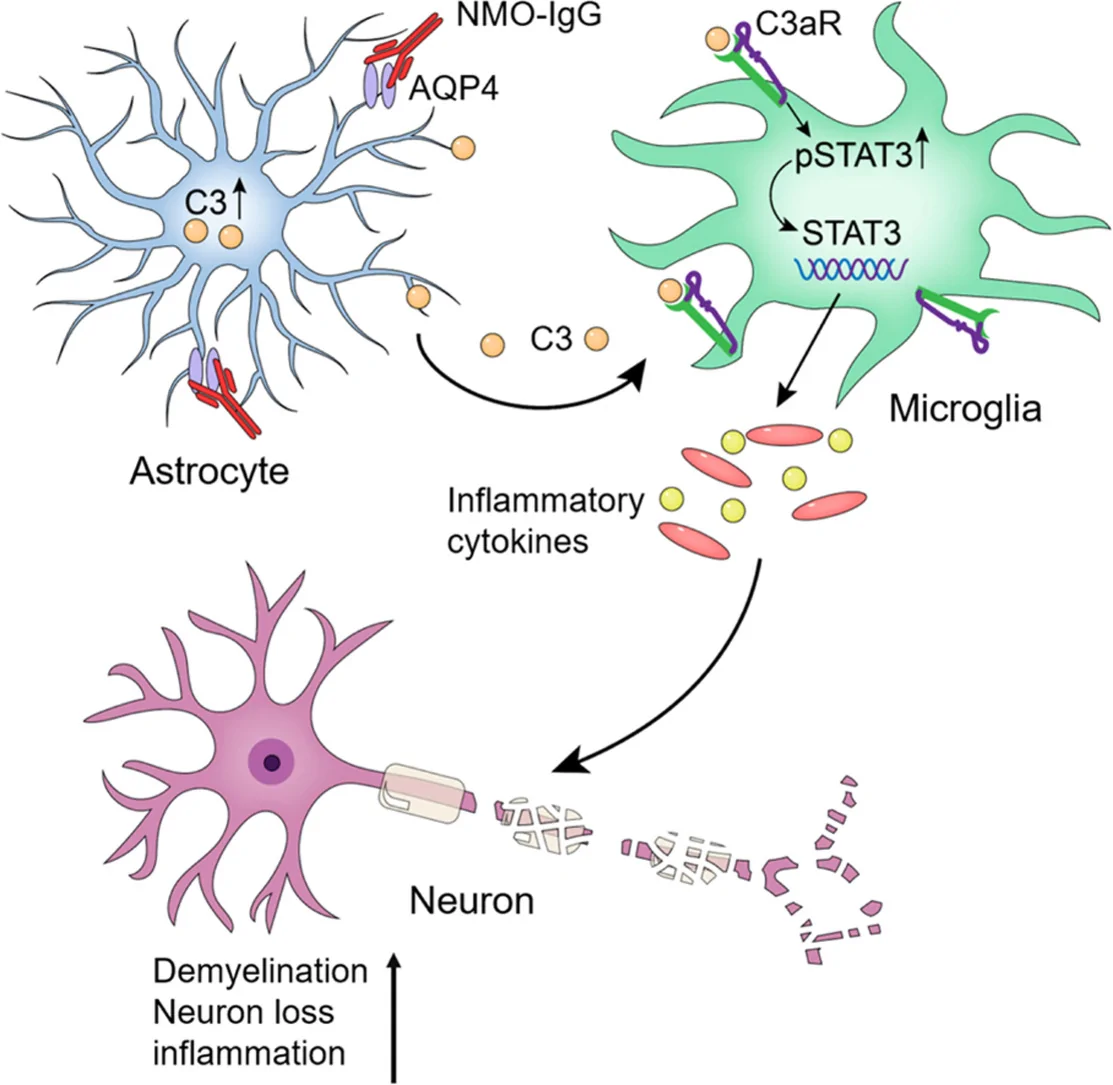

## 1 Introduction

Neuromyelitis optica (NMO) is defined as an autoimmune inflammatory disease characterized by prominent demyelination in specific tissues of the central nervous system (CNS), causing corresponding neurological defects in cognition, vision, and motion [1]. Inflammatory overreaction is recognized as essential to the injury of astrocytes, oligodendrocytes, and neurons during NMO progression, primarily manifesting as microgliosis, complement deposition, and hyperaccumulation of proinflammatory cytokines [2]. In NMO, the antibody and complement systems play vital roles in the pathogenesis of neuroinflammation. Specifically, pathogenic antibodies, such as NMO-IgG, specifically bind to the astrocytic aquaporin 4 (AQP4) water channel, initiating a cascade of immune responses [3]. This binding event leads to the activation of the complement system, which is a key component of the innate immune system. The complement cascade, particularly the C3 activation pathway, is a key mediator of neuroinflammation across NMO and other autoimmune and degenerative CNS disorders. Dysregulated complement activation drives neuronal damage through opsonization (C3b), inflammatory signaling (C3a/C5a), and membrane attack complex (MAC) formation, with microglia and astrocytes serving as major cellular effectors [4]. Notably, in peripheral autoimmune diseases like rheumatoid arthritis and lupus, macrophage-C3aR interactions critically amplify inflammation by enhancing proinflammatory cytokine release (e.g., interleukin-1β (IL-1β), tumor necrosis factor-α (TNF-α)) and impairing regulatory functions [5,6]. This underscores the conserved role of C3aR in innate immune dysregulation. Microglia, as CNS-resident macrophages, share functional parallels with peripheral macrophages in complement responses. Recent evidence indicates that C3aR signaling in macrophages promotes proinflammatory polarization in autoimmune conditions [[5], [6], [7]], providing a framework to explore analogous mechanisms in microglial-driven neuroinflammation. Indeed, neuroinflammation induced by activated microglia has gained growing attention in various neurological disorders, such as Alzheimer’s disease (AD) [8], amyotrophic lateral sclerosis (ALS) [9], and multiple sclerosis (MS) [10]. Studies strongly support the pathogenic role of microglia activation in NMO [[11], [12], [13]].

Microglia, as the resident immune cells of the CNS, play an essential role in neuroinflammatory diseases, partly through participating in the classical complement activation process, thereby regulating innate immunity [[14], [15], [16]]. The key step to trigger the classical complement cascade is the cleavage of the central complement factor C3 into C3a and C3b, combining with their receptors C3aR and CR3, respectively [17]. Recent studies have revealed that C3-C3aR signaling not only directly contributes to neuronal damage but also exacerbates neuroinflammation [8,11,18]. C3aR expression has been found to be upregulated on activated microglia upon these inflammatory conditions [8,11,18], suggesting a close association between C3aR expression and microglia activation in promoting neuroinflammation. In addition, activated microglia-derived complement component 1q (C1q) initiates complement activation and axonal injury in neurodegenerative diseases [19,20]. In NMO, a study found that NMO-IgG promotes microglial C1q production in an NMO mouse model with dyskinesia due to motor neuron loss [11]. Activated microglia exhibit dynamic states ranging from “proinflammatory” (producingIL-1β, IL-6, and TNF-α), while suppressing the release of anti-inflammatory factors (IL-10, IL-4, IL-13, and transforming growth factor-β (TGF-β)) to “alternatively activated” phenotypes (associated with tissue repair); this continuum influences disease outcomes in NMO [[21], [22], [23]]. However, it remains unclear whether this is due to C3aR signaling mediating microglial activation to produce these cytokines.

NMO pathogenesis begins with NMO-IgG binding astrocytes, followed by microglia activation [1,2]. Some previous studies demonstrated that NMO-IgG could induce astrocytic C3 production, and targeting inhibition of C3 attenuates microglial activation and reduces the contents of proinflammatory cytokines (IL-1β, IL-6, C1q, and TNF-α) in NMO mouse models [24]. Similarly, other researchers have shown that genetic C3aR deletion on microglia reduces activation and C1q production, ameliorating NMO pathology and motor dysfunction in vivo [11]. Thus, C3aR emerges as a critical node in glial interactions driving NMO. However, the mechanism by which C3aR signaling activates microglia to promote inflammation during NMO progression remains unclear.

To investigate the role of C3aR in NMO, we analyzed its expression on microglia across complementary experimental models: in vitro, *ex vivo*, and in vivo. Our results demonstrated a significant upregulation of microglial C3aR concomitant with pronounced microglial activation and elevated production of proinflammatory factors during the acute phase of NMO pathogenesis. Consistently, both pharmacological inhibition of C3aR and lentivirus-mediated C3aR knockout proved effective in attenuating microgliosis and suppressing the secretion of proinflammatory cytokines. These interventions conferred a protective effect against NMO-associated demyelination and neuronal damage and ameliorated key clinical manifestations, including visual impairment, limb disability, and cognitive deficits, in vivo models of NMO. Mechanistically, we identified signal transducer and activator of transcription 3 (STAT3) as a critical downstream effector of C3aR signaling in microglia. Importantly, inhibition of STAT3 phosphorylation recapitulated the beneficial effects, mitigating NMO-related pathology and functional impairments.

## 2 Materials and methods

### 2.1 Cell lines

BV2 cells (CVCL_0182) were maintained in Dulbecco’s Modified Eagle’s Medium (DMEM, 10569010, GIBCO) supplemented with 10% Fetal Bovine Serum (FBS, ST30-3302, PAN™ SERATECH) and cultured at 37 °C in a 5% CO_2_ atmosphere. Human embryonic kidney (HEK293T) cells (ATCC, CRL-11268) were maintained in Dulbecco’s modified Eagle medium (DMEM) supplemented with 10% FBS and 1% penicillin-streptomycin (PS, 15140-122, GIBCO) under the same conditions.

### 2.2 Primary astrocyte isolation and culture

Primary astrocytes were isolated and cultured as previously described [24]. Briefly, brains (excluding bilateral hippocampi) from P0 mouse pups were washed with DMEM-F12 (C11330500BT, GIBCO) containing 10% FBS and 1% PS (to make DMEM-F12 complete medium). The brains were then digested with 0.25% trypsin-EDTA (25200056, GIBCO) for 20 min, with trypsinization halted by DMEM-F12 complete medium. After centrifugation at 1000 g for 5 min, the single-cell suspension was collected and cultured in T-75 flasks at 37 °C in a 5% CO_2_ atmosphere for 14 days using astrocyte proliferation medium. This medium included DMEM-F12 supplemented with 10% FBS, 1% non-essential amino acids (NEAA, 11140050, GIBCO), 1% glutamine (25030-081, GIBCO), 1 mM sodium pyruvate (S8636, Sigma), and 1% PS, with medium replacement every 2–3 days. Astrocytes were characterized by GFAP staining (1:1000, 12389S, CST) prior to use.

### 2.3 Experimental animals

Wild-type C57BL/6 mice and Sprague-Dawley (SD) rats were obtained from Vital River Company. They were housed at 4–5 per cage with *ad libitum* access to food and water, under a 12-h light/dark cycle at the Guangdong Laboratory Animals Monitoring Institute. Female mice, aged 8 weeks, and female rats, weighing between 230 and 250 g, were utilized for the experiments. All animal experiments comply with the ARRIVE guidelines and are carried out in accordance with the National Research Council’s Guide for the Care and Use of Laboratory Animals. This study received approval from the Institutional Animal Care and Use Committee of Sun Yat-sen University (No. SYSU-IACUC-2023-B0123).

### 2.4 Human IgG purification

IgG purification followed the protocols from our prior study [24]. Initially, plasma was collected from both seropositive NMO patients and healthy volunteers. Total IgG was purified using protein A beads (71149800-EG, GE Healthcare, USA), eluted with 0.1 M glycine-HCl (pH 2.5), and concentrated using Amicon Ultra15 centrifugal filter units (100 kD, Millipore, Billerica, MA). The eluent was then sterilely filtered through a 0.22 µm filter. The resulting working IgG concentrations were designated as NMO-IgG (26.5–30.5 mg/mL) and Con-IgG (25.5–32.0 mg/mL), and stored at −80 °C. Human complement (HC) was sourced from Innovative Research (Novi, MI, USA). Informed consent was obtained from all participants, with approval by the Ethics Committee of the Third Affiliated Hospital of Sun Yat-sen University (No. 02-405-01).

### 2.5 Lentivirus packaging

Lentivirus packaging was executed as previously detailed [43]. Target and packaging plasmid DNAs were co-transfected into 293T cells using polyethyleneimine (PEI, PR40001, Proteintech Group). The medium was replaced 4 h post-transfection and harvested at 40, 64, and 88 h, then filtered through a 0.22 µm filter (BS-QT-037, Biosharp). Viral particles were concentrated by ultracentrifugation (SW32 Ti, Beckman) at 20,000 rpm for 2 h at 4 °C, washed with Dulbecco’s phosphate-buffered saline (DPBS, 14190136, GIBCO), and resuspended in 100 µL DPBS. Viruses were aliquoted and stored at −80 °C. The estimated viral titer was approximately 1 × 10^12^ particles per milliliter. Primers used were as follows:

- C3 shRNA: 5′-GCCCGTGATTCACCAAGA AAT-3′
- C3aR shRNA: 5′-CCCGTATTTGTATACCGTGAT-3′

### 2.6 Primary astrocytes and BV2 cells in vitro assay

Primary astrocytes were transfected with Lenti-shC3 or Lenti-shNC virus for 48 h, followed by treatment with NMO-IgG at a concentration of 100 ng/mL for 24 h. The conditional medium was collected for BV2 cell assays, and astrocytes were lysed for WB analysis. BV2 cells were seeded at 1 × 10^4^ on coverslips in 24-well plates and treated with human C3 protein (P02621, Solarbio) at 1 µg/mL, phosphate-buffered saline (PBS, C10010500BT, GIBCO), or conditional medium from astrocytes for 24 h. The conditional medium was collected at 1 h, 4 h, and 24 h for ELISA analysis. Cells were fixed with 4% paraformaldehyde (PFA, 158127, Sigma) for immunostaining and lysed for WB analysis, respectively.

### 2.7 *Ex vivo* model of organotypic cerebellar slice cultures

Organotypic cerebellar slices were prepared as previously described [24]. The whole cerebellum was rapidly removed from P7 mouse pups and placed in ice-cold Hank’s Balanced Salt Solution (HBSS, pH 7.2, Invitrogen, USA). The cerebellum was then sliced into 350 µm thick transverse sections using the McIlwain Tissue Chopper (Cavey Laboratory Engineering, England). Slices were placed on transparent, non-coated membrane inserts in 6-well plates (Corning 3450 Transwell, 0.4 µm pores) and cultured in specific medium at 37 °C in a 5% CO_2_ atmosphere for 7 days. The culture medium consisted of 50% Minimum Essential Medium (MEM, 42360032, GIBCO), 25% HBSS, 25% horse serum (SH30074.02, HyClone), 200 mg/mL d-glucose, 2 mM glutamine, and 1% PS. The medium was changed the day after seeding and subsequently every 2 days. Slices received a mixture of NMO-IgG or Con-IgG at a concentration of 1mg/mL and 10% HC with or without 10 µM SB (HY-101502A, MCE) or SH-4-54 (KM11475, KKL Med), using dimethyl sulfoxide (DMSO, D103273, Aladdin) as the vehicle. After a 48-h incubation, the culture medium of each group was collected for Elisa analysis. And slices were washed with cold PBS and fixed in 4% paraformaldehyde (PFA) for immunostaining. The severity of NMO-like lesions in cerebellar slices was assessed based on AQP4 and GFAP markers, with scoring as follows: 0, normal GFAP and AQP4 staining; 1, decreased AQP4 staining with or without mild astrocyte swelling; 2, reduced GFAP and AQP4 staining in at least one lesion; 3, > 30% of the slice area affected by multiple lesions; and 4, > 80% of the slice area affected by multiple lesions [44].

### 2.8 Focal intracerebral injection mouse model of NMO

This model was established as previously described [24]. Mice were anesthetized with isoflurane and secured in a stereotaxic frame (RWD Life Sciences, Shenzhen, China). An incision was made between the ears to expose the skull, and coordinates for the corpus striatum were marked: 0.5 mm anterior to the bregma, ±2.0 mm lateral, and −3.0 mm ventral. A 1 mm diameter hole was drilled on each side, and a mixture containing 3 µL of NMO-IgG or Con-IgG, 2 µL of HC, and 1 µL of Lenti-shC3aR or Lenti-shNC was infused into each site using a 26-gauge needle attached to a 10 µL gas-tight glass syringe (Hamilton) over 6 min. The needle was left in place for an additional 10 min before being slowly withdrawn. Mice were then returned to their housing cage on a heating pad until they fully recovered.

### 2.9 Optic neuritis rat model of NMO

The optic neuritis model was based on our previous study [24]. Rats were anesthetized with isoflurane, and the optic nerve was exposed via blunt dissection from the superior conjunctiva. NMO-IgG or Con-IgG (6 µL) was injected beneath the optic nerve sheath, approximately 2 mm posterior to the globe, using a 33-gauge needle attached to a 10 µL gas-tight glass syringe (Hamilton). The needle was left in place for an additional minute after injection. Care was taken to minimize damage to the ON or retina. Antibiotic ointment was applied post-operation. SB (10 mg/kg) or SH-4-54 (20 mg/kg) was administered intraperitoneally on days 2, 4, and 6 post-injections, with corn oil used as the vehicle.

### 2.10 Systemic mouse model of NMO

The systemic mouse model of NMO was conducted according to our previously described methods [24]. To compromise the blood-brain barrier (BBB), mice were initially injected subcutaneously at four sites on the hindquarters with a solution containing complete Freund’s adjuvant (CFA, Sigma-Aldrich) and heat-killed H37Ra *Mycobacterium tuberculosis* (MTB, BD-DIFCO) at a dosage of 50 µg per site, administered once, 7 days before IgG injection. Additionally, mice received a single intraperitoneal injection of pertussis toxin at a dose of 200 ng per mouse (PTX, Enzo Life Sciences) on days 7 and 4 prior to IgG transfer, respectively. Subsequently, mice were injected intraperitoneally once with NMO-IgG or Con-IgG at a concentration of 20 mg/mL for a continuous period of 10 days. Concurrently, some mice in the NMO group were also administered intraperitoneal injections of SB (1 mg/kg) [45] or SH-4-54 (10 mg/kg) [8] on days 2 and 6 following NMO-IgG administration.

### 2.11 Balance beam (BB) test

The BB test was conducted as described previously [31]. The apparatus consisted of a beam measuring approximately 0.6 cm in width and 100 cm in length, suspended 50 cm above several foam pads. A lamp positioned at the beam’s starting point served as the stimulus, while a black box at the end of the beam acted as the goal. Mice were trained to traverse the beam once daily for 2 days prior to the test. During the test, the number of paw slips and the time taken to cross the beam were recorded as measures of performance.

### 2.12 Novel object recognition (NOR) test

The NOR test procedure was carried out in accordance with a previously published study [43]. Mice underwent training 2 days before the test. On the first day, mice were allowed 10 min of free exploration in an empty chamber. The following day, they explored two identical objects (cylinders) placed parallel to each other for 10 min. On the test day, each mouse was placed in the same chamber with two different objects (one cylinder and the other replaced with a triangular prism) positioned at the same locations as previously and allowed to explore for 10 min. The exploratory preference for the novel object was defined as the percentage of time spent investigating the novel object out of the total time spent exploring both objects.

### 2.13 Electrophysiological test

Flash visual evoked potentials (F-VEP) and electroretinography (ERG) were utilized to examine the visual function of rat models with NMO-related optic neuritis. Electrophysiological tests were conducted 1 day prior to surgery and on days 3 and 7 after injection, as described previously [24]. For the VEP test, positive electrodes (stainless steel screws) were implanted into the skull 7 mm posterior to the bregma and 3 mm lateral to the midline above the visual cortex area using a stereotaxic device. The reference electrode was implanted in the midline region 3 mm anterior to the bregma, and a needle electrode for the ground was inserted into the tail [46]. Prior to VEP recording, rats were acclimated to darkness for 2 h, then anesthetized with 2% pentobarbital sodium in a dark room under dim red light. Pupils were dilated with one drop of compound tropicamide (0.5% tropicamide and 0.5% epinephrine hydrochloride, Xing Qi Ophthalmic Co., Ltd., Shenyang, China), and corneal dryness was prevented with intermittent application of artificial tears. The skin on the skull was opened to attach screws to the amplifier. Visual stimulation (9.49 cd*s/m^2^) comprised 1 Hz white light flashes, produced by a full-field Ganzfeld stimulator (Roland Consult, Brandenburg, Germany) in dark-adapted conditions. A hundred flash stimuli were repeated three times at 5-min intervals. Each eye was recorded separately while the opposite eye was covered with a homemade black visor. Upon completion, antibiotics were applied topically after closing the skin on the skull. Signals were filtered between 0.5 Hz and 50 Hz, with data collected via Roland software (Roland Consult, Brandenburg, Germany). The P1 latency was defined as the time from light onset to the first positive wave peak, and the P1 amplitude was recognized as the difference from the trough of the first negative peak after light onset (N1) to the peak of P1. Each eye was measured at least three times to obtain an average value.

For the ERG test, rats were acclimated to overnight darkness before recordings. They were then anesthetized with 2% pentobarbital sodium, with pupils dilated and lubricated with 1% methylcellulose. Two positive electrodes were carefully placed on the corneal surface, and two reference electrodes were inserted into the hypodermis between the ears. A needle electrode served as the ground electrode in the tail. Retinal potential was evoked by flickering light stimuli from the ERG recording system (Roland Consult, Brandenburg, Germany). Light acclimatization lasted for 10 min against a white background of 25 cd/m^2^ to inhibit rod cell photosensitivity. Photopic negative responses (PhNR) were recorded with white flashes of 3 cd/m^2^ or 10 cd/m^2^ at a stimulus frequency of 0.03 Hz. Each eye was recorded individually while the opposite eye was covered with a homemade black visor. The PhNR amplitude was defined as the amplitude from the largest negative wave following the baseline to the b-wave. All statistical data were presented as absolute values.

### 2.14 Motor behavior test

Motor behavior tests were conducted as previously described [24]. Following IgG injection, mice underwent behavioral assessments starting from day 0 and every other day until day 10. In the rotarod task, each mouse was placed on a cylinder that began rotating at 10 revolutions per minute (rpm), gradually increasing to 40 rpm within 5 min. This task was repeated three times per mouse with 15-min intervals, and the average latency to fall was recorded from three attempts. For the gait analysis, mice were observed walking across a narrow strip, and their performance was captured by the Catwalk-assisted gait analysis system (XR-FP101, SHXINRUAN Corporation, China). The median stride length of the hindlimbs was calculated from five sequential steps.

### 2.15 Immunostaining and image analysis

After intracardiac perfusion with 4% PFA, mouse brains and spinal cords were post-fixed overnight at 4 °C in 4% PFA and subsequently transferred into a 30% sucrose solution. Brain coronal sections and lumbar-4 spinal cord transverse sections were sliced to a thickness of 40 µm using a microtome (Thermo Fisher Scientific, USA) and preserved in cryoprotectant at 4 °C. Following anesthetization and intracardiac perfusion with 0.9% saline and 4% PFA in sequence, the entire optic nerve was extracted, then fixed at 4 °C for 1 h, thoroughly rinsed with PBS, and dehydrated in 0.01 M PBS containing 15% and 30% sucrose, respectively. Each optic nerve was embedded in an optimal cutting temperature compound (OCT; Tissue Tek, Torrance, CA, USA) and longitudinally sectioned to a thickness of 10 µm using a microtome (Leica Microsystems, Buenos Aires, Argentina). All samples were mounted on glass slides and stored at −80 °C.

For immunofluorescence staining, cerebellar slices were initially rinsed three times with PBS following fixation in 4% PFA for 1 h at room temperature (RT). Subsequently, slices were permeabilized with 0.5% Triton X-100 for 30 min, followed by incubation in blocking buffer A, which consists of 10% donkey serum (Sigma‒Aldrich, D9663) or goat serum (16210072, Gibco) in PBS for 1 h at RT. Primary antibodies were applied overnight at 4 °C. BV2 cells on coverslips, along with brain, spinal cord, and optic nerve sections, were blocked with blocking buffer B comprising PBS with 3% donkey or goat serum and 2.5% Triton X-100 for 1 h at RT, followed by overnight incubation with primary antibodies at 4 °C. The primary antibodies used included mouse anti-C3aR (1:100, Santa Cruz Biotechnology), rabbit anti-AQP4 (1:1000, Santa Cruz Biotechnology), mouse anti-GFAP (1:1000, CST), rabbit anti-Iba1 (1:1000, Wako Chemicals), rat anti-CD68 (1:1000, Abcam), goat anti-C3d (1:300, R&D), rabbit anti-C5b-9 (1:500, Abcam), rabbit anti-MBP (1:500, CST), chicken anti-NFH (1:400, Millipore), mouse anti-NeuN (1:500, Abcam), rabbit anti-STAT3 (1:500, CST) and mouse anti-pSTAT3 (1:500, CST). Following several PBS washes, sections or coverslips were incubated with fluorescent secondary antibodies for 1 h at RT and mounted with polyvinyl alcohol (PVA, 02815, Polysciences). The fluorescent secondary antibodies utilized were goat anti-mouse 488 (1:1000, Invitrogen), goat anti-mouse 568 (1:1000, Invitrogen), goat anti-chicken 488 (1:1000, Invitrogen), goat anti-chicken 647 (1:1000, Invitrogen), goat anti-rabbit 488 (1:1000, Invitrogen), goat anti-rabbit 568 (1:1000, Invitrogen), goat anti-rabbit 647 (1:1000, Invitrogen), donkey anti-goat 488 (1:1000, Invitrogen), donkey anti-goat 647 (1:1000, Invitrogen), and donkey anti-rat 568 (1:1000, Invitrogen). Nuclei were stained with 4′,6-diamidino-2-phenylindole (DAPI, D2261, Sigma Aldrich). BV2 cells on coverslips, select cerebellar slices, and original brain sections were imaged at 10x magnification using an upright fluorescent microscope (Nikon, Eclipse E100, Japan). Other samples were imaged using single-plane and z-stack confocal microscopy at 10x and 20x magnification (Leica, TCS SP8, Germany). Each experiment was replicated a minimum of three times.

The evaluation of NMO-like lesions across different models referred to our previously reported study [24]. In assessing brain lesions, we compared the positive area of Iba1, C3aR, CD68, C3d, and C5b-9, alongside the loss area of GFAP, AQP4, MBP, and NFH among different mouse groups. Conversely, in other models, we quantified Iba1^+^microglia per cubic millimeter for intergroup comparisons. Additionally, we measured the number of NeuN^+^ neurons, the percentage of C3aR or CD68 positive Iba1^+^ microglia, and the nuclear/cytoplasmic ratio of p-STAT3 in different models. Remaining images were analyzed for relative fluorescence intensities of GFAP, AQP4, MBP, NFH, C3d, and C5b-9 to discern differences among groups.

### 2.16 Quantification of retinal ganglion cells

Retinal ganglion cells (RGCs) were counted following the procedure described previously [24]. Whole-mount retinas from rats were fixed in 4% PFA for 1 h and washed with PBS. The samples were then blocked with CAS-Block blocking buffer (Life Technologies, USA, 00-8120) containing 3% Triton-X-100 for 1 h at RT, followed by incubation with rabbit anti-RBPMS antibody (1:500, Santa Cruz Biotech) for 48 h at 4 °C. After several PBS washes, retinas were incubated with secondary antibodies, donkey anti-rabbit 647 (1:1000, Invitrogen), for 2 h at RT and mounted with PVA. The number of RBPMS-positive cells, indicative of surviving RGCs, was visualized using a laser confocal microscope (Leica, TCS SP8, Germany). Each retina was divided into four quadrants; in each, three images of 0.06 mm^2^ were captured from peripheral, medial, and central retina sections, each 1 mm apart. An average of 12 images was calculated for each retina [47].

### 2.17 RGC axonal counting

Axonal counting was conducted as described [48]. Optic nerves were initially fixed in ice-cold 5% glutaraldehyde and 0.1 M PBS solution at 4 °C for 48 h, then immersed in 1% osmium tetroxide (pH 7.4) for 3 h. This was followed by dehydration in ethanol (75%, 95%, and 100%) and embedding in epoxy resin. Coronal semi-thin sections of 1 µm thickness were obtained, dried on slides, and stained with 1% toluidine blue (Sigma-Aldrich). Images from 3 to 5 sections (2–3 mm from the eye globe’s distal end) for each optic nerve were captured at 10x and 63x magnification using a bright-field microscope (Nikon, Eclipse E100, Japan). Complete coronal optic nerve sections were scored on a scale of 1 to 5 by three independent researchers, categorized as healthy (grade 1), moderate (grades 2 and 3), or severe damage (grades 4 and 5) [49]. Axonal survival was quantified using automated counting in ImageJ and defined as the density of axons per 1000 µm^2^.

### 2.18 Electron microscopy and G-ratio determination

Electron microscopy observation was performed following previous methodology [24]. Rats were perfused with glutaraldehyde fixative, and ONs were sectioned into 1 mm^3^ pieces and post-fixed in 2.5% glutaraldehyde. After washing, tissues were fixed in 1% osmium tetroxide in 0.1 M PBS (pH 7.4) for 2 h at RT, dehydrated in graded ethyl alcohol and acetone, and embedded in EMBed 812. Transverse ultrathin sections (60–80 nm) were stained with uranyl acetate and lead citrate, examined under an HT7800 transmission electron microscope (HITACHI, Japan). The G-ratio was analyzed using ImageJ, defined as the ratio between axon and fiber diameter.

### 2.19 Western blot analysis

Biological samples were lysed in radioimmunoprecipitation assay (RIPA) buffer with protease and phosphatase inhibitors. Protein concentration was determined using a bicinchoninic acid (BCA) protein assay kit (KeyGEN BioTECH, KGP902). Proteins were separated by 10% sodium dodecyl sulfate-polyacrylamide gels (SDS-PAGE), transferred to ethanol-activated (polyvinylidene fluoride) PVDF membranes, blocked in 5% skim milk, and incubated with primary antibodies (mouse anti-C3aR and mouse anti-p-STAT3, 1:1000, CST) overnight at 4 °C. Following TBST washes, membranes were incubated with anti-mouse HRP-conjugated secondary antibody (1:5000, HuABio) for 1 h at RT, with proteins visualized using SuperSignal™ West Pico PLUS chemiluminescent substrate (Thermo Scientific, 34580, USA). Protein intensity was normalized to rabbit anti-β-actin (HuABio, HA722023) and quantified with Image J.

### 2.20 ELISA assay

Levels of inflammatory factors (IL-1β, IL-6, IL-10, C1q, and TNF-α) in conditional media of BV2 cells or organotypic cerebellar slices, as well as mouse spinal cord tissue homogenates, were measured using ELISA kits (Jiangsu Meimian Industrial Co., Ltd, China) according to the manufacturer’s instructions.

### 2.21 Statistical analysis

G-ratio values and axonal size distributions were presented as scatter plots. Statistical analyses were conducted using GraphPad Prism, with data expressed as mean ± standard error (SEM). Normality was assessed with the Shapiro-Wilk test. Two-tailed Student’s *t*-tests and one-way analysis of variance (ANOVA) with Tukey’s post hoc test were used for comparisons, and optic nerve degeneration severity was analyzed by the Kruskal-Wallis test. P1 latency and amplitude in VEP tests, PhNR amplitude in ERG tests, and rotarod test performance were analyzed by two-way ANOVA with Tukey’s post-test. Significance thresholds were set at *p < 0.05, ** *p* < 0.01, ** *p < 0.001, ****p < 0.0001.

## 3 Results

### 3.1 Inhibiting C3aR signaling ameliorates the inflammatory response in organotypic cerebellar slice models of NMO

Astrocyte-microglia interaction plays a vital role in many CNS diseases, such as AD, hydrocephalus, ischemic stroke, and so on [[25], [26], [27]]. As previously reported, astrocytes increase C3 production induced by NMO-IgG, accompanied by microglial activation [24]. The activation fragments of C3a from the cleavage of C3, which elicit downstream events through binding to its receptor C3aR, are one of the requirements to activate the classical complement pathway [17]. Therefore, we speculated that microglia are activated by C3aR binding to C3 in NMO pathogenesis. To verify this hypothesis, we first examined the expression of C3aR in BV2 cells stimulated by human C3 protein (Fig. 1a). Compared with BV2 cells treated with PBS, both the C3aR protein level and the percentage of C3aR^+^ BV2 cells were significantly increased after C3 protein treatment (Fig. 1b,c), indicating that C3aR is upregulated in microglia after C3 binding. Moreover, we introduced a lentivirus (LV) coexpressing green fluorescent protein (GFP) with C3 shRNA (Lenti-shC3) or control shRNA (Lenti-shNC) into primary mouse astrocytes to abrogate C3, and the C3 knockdown efficiency was confirmed by western blot (WB) (Fig. S1a,b). Then, we treated BV2 cells with the supernatant of astrocytes after 24 h of NMO-IgG stimulation (Fig. S1c). As expected, the percentage of C3aR^+^ BV2 cells was significantly decreased in the Lenti-shC3 astrocytic supernatant group compared with the Lenti-NC astrocytic supernatant group (Fig. S1d), suggesting that the expression of microglial C3aR is regulated by astrocytic C3 in NMO. Activated microglia have two phenotypes: “proinflammatory” type produces proinflammatory cytokines (C1q, IL-1β, and TNF-α, etc.) and “alternatively activated” type produces anti-inflammatory cytokines (IL-10, IL-4, etc.) [21,22,28]. To further evaluate the influence of microglial C3aR upregulation on the inflammatory reaction, we collected the supernatant of BV2 cells 1h, 4h, and 24h after C3 protein stimulation to examine the contents of cytokines (IL-1β, TNF-α, C1q, and IL-10) released by microglia. Compared to the supernatant of BV2 cells treated with PBS, the contents of proinflammatory cytokines (IL-1β, TNF-α, and C1q) were higher in the supernatant of BV2 cells after 24 h of C3 protein stimulation, while the content of the anti-inflammatory cytokine IL-10 was lower in the supernatant as early as 1h after C3 protein stimulation (Fig. 1d). These results demonstrated that microglia transition to a proinflammatory phenotype amplifies the inflammatory response after C3-C3aR axis activation.

**Fig. 1:**
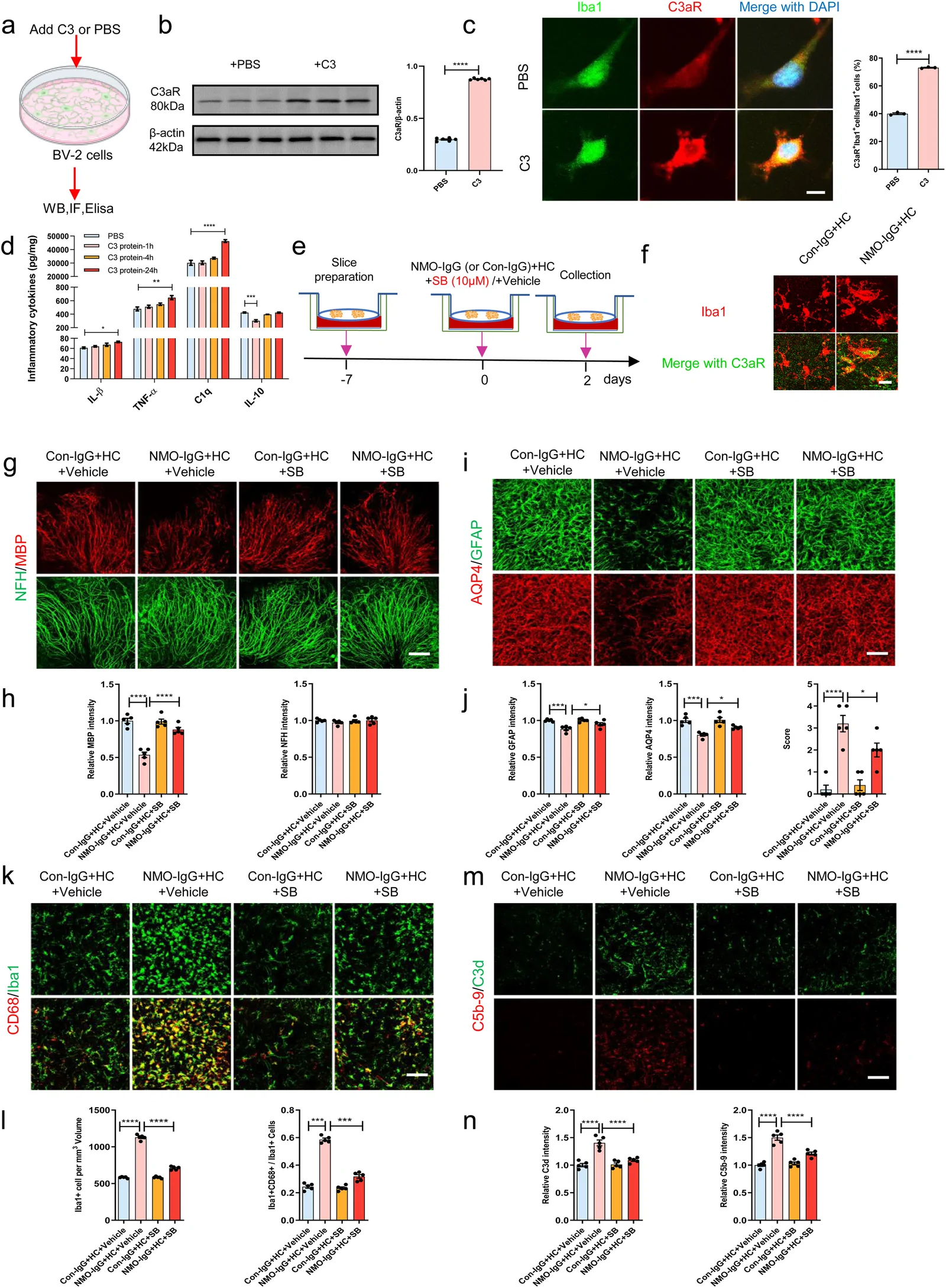
**Impact of C3aR upregulation and C3aR antagonism on microglial activation and NMO-like lesions**. (a) BV2 cell assay schematic. (b) Representative immunoblots (left) and quantification of C3aR levels (right) in BV2 cells after 24 h PBS or human C3 protein treatment. *n* = 6 biological replicates for each group. (c) Iba1 (green) and C3aR (red) co-staining with DAPI (blue) in treated BV2 cells, showing C3aR^+^ Iba1^+^ microglia percentage across groups. Scale: 20 µm. *n* = 3 slides (6 sections per slide)) for each group. (d) Inflammatory cytokines (C1q, IL-1β, TNF-a and IL-10) quantification in the culture medium of treated BV2 cells at 1 h, 4 h, and 24 h across groups. *n* = 3 biological replicates for each group. (e) NMO cerebellar slice model experimental timeline with SB or DMSO treatment. (f) Iba1 (red) and C3aR (green) co-staining in cerebellar slices post 48 h treatment of NMO-IgG and HC or Con-IgG and HC. Scale: 50 µm. (g) MBP (red) and NFH (green) staining in cerebellar slices across groups. Scale: 100 µm. (h) MBP and NFH relative immunofluorescence intensity in cerebellar slices across groups. *n* = 5 slices (3 sections per slice) for each group. (i) GFAP (green) and AQP4 (red) staining in cerebellar slices across groups. Scale: 100 µm. (j) GFAP, AQP4 relative fluorescence intensity, and NMO-like lesion scoring in cerebellar slices across groups. *n* = 5 slices (3 sections per slice) for each group. (k) Iba1 (green) and CD68 (red) co-staining in cerebellar slices across groups. Scale: 100 µm. (l) Iba1^+^ microglia count and CD68^+^ Iba1^+^ microglia percentage in cerebellar slices across groups. *n* = 5 slices (3 sections per slice) for each group. (m) C3d (green) and C5b-9 (red) staining in cerebellar slices across groups. Scale: 100 µm. (n) C3d and C5b-9 relative fluorescence intensity in cerebellar slices across groups. *n* = 5 slices (3 sections per slice) for each group. Data are shown as mean ± SEM, analyzed via Student’s *t*-test for (b, c, f) and one-way ANOVA with Tukey’s post hoc for other comparisons. *p < 0.05, **p < 0.01, ***p < 0.001, ****p < 0.0001. Non-significant results omitted.

Since inflammatory overreaction is the basis of NMO pathology [2], we next studied whether targeting the inhibition of C3aR could reduce inflammation to ameliorate NMO-like lesions in organ slice culture models of NMO. As described in our previous study [24], we prepared cerebellar slices from postnatal 7-day-old (P7) mice. After 7 days of preculturing, we added 10 µM SB290157 trifluoroacetate (SB), identified as a selective, high-affinity, competitive antagonist of C3aR in many studies [25,26,29,30], to slices treated with 1 mg/mL NMO-IgG (or Con-IgG) and 10% human complement (HC) for 48 h (Fig. 1e). Consistent with cellular results, we found a remarkable increase in microglial C3aR expression in cerebellar slices treated with NMO-IgG and HC (NMO group) compared to those treated with Con-IgG and HC (Control group) (Fig. 1f), indicating that C3aR expression was strikingly upregulated in the presence of NMO-IgG. As expected, SB treatment significantly recovered the loss of myelin, reduced astrocyte damage, and preserved axons in NMO group slices, as shown by increasing the immunofluorescence intensity of myelin basic protein (MBP, a myelin marker), AQP4, and glial fibrillary acidic protein (GFAP, an astrocyte marker), while the immunoreactivity level of neurofilament H (NFH, an axonal marker) did not exhibit a significant change among different groups (Fig. 1g–j). Importantly, both the number of ionized calcium-binding adaptor molecule 1 (Iba1)-positive microglia and the percentage of CD68^+^ Iba1^+^ microglia (activated microglia) were robustly decreased in the SB treatment group slices compared to NMO group slices (Fig. 1k,l), suggesting that inhibiting C3aR signaling with SB could effectively reduce microglial aggregation and activation in *ex vivo* NMO models. Along with this, the protein levels of C1q, IL-1β, and TNF-α had a marked reduction in the medium of NMO slices with SB treatment, while that of IL-10 had an elevation (Fig. S1e). Given that C1q, produced by microglia, is a component of the complement system, we examined the deposition of C3d (C3 activation fragments) and C5b-9 (the complement terminal complex) in slices. Similarly, SB treatment reversed the higher immunoreactivity levels of C3d and C5b-9 in NMO group slices (Fig. 1m,n), suggesting that SB also has a negative regulatory effect on complement activation. Taken together, these observations demonstrated that SB treatment alleviated NMO-like lesions by inhibiting C3aR to relieve the inflammatory response in an *ex vivo* NMO model.

### 3.2 Lenti-shC3aR could reduce microglial activation to alleviate NMO pathological lesions and neurological impairment in a focal intracerebral injection mouse model of NMO

To further investigate the therapeutic role of C3aR in NMO, we first found that C3aR was mainly expressed on microglia in the corpus striatum of a focal intracerebral injection mouse model of NMO through immunofluorescence co-staining (Fig. S2a–e). Then, we reduced C3aR expression in the corpus striatum using an in vivo virus-mediated RNA interference (RNAi) approach. We first constructed lentiviruses (LVs) expressing GFP with C3aR shRNA (Lenti-shC3aR) and verified their high efficiency in downregulating C3aR protein synthesis in HEK293 cells (Fig. 2a,b). Then, as previously described in our study [24], we performed bilateral, intracerebral injections of Lenti-C3aR (or Lenti-NC) with NMO-IgG (or Con-IgG) and HC into the corpus striatum of 8-week-old adult wild-type C57BL/6 mice (Fig. 2c). To assess neurological function associated with the corpus striatum, mice were subjected to the balance beam (BB) test and the novel object recognition (NOR) test 1 week after injection, as reported in a previous study (Fig. 2d) [31]. Compared with the control group, mice in the NMO group had a higher frequency of paw slips during the test without spending more time crossing the BB (Fig. 2e,f), suggesting that NMO mice suffer from motor balance and coordination dysfunction, but not from physical disability. Moreover, we also found that mice in the NMO group showed less preference for the location of the novel object in the NOR test (Fig. 2g,h), implying a deficit in learning and memory.

**Fig. 2:**
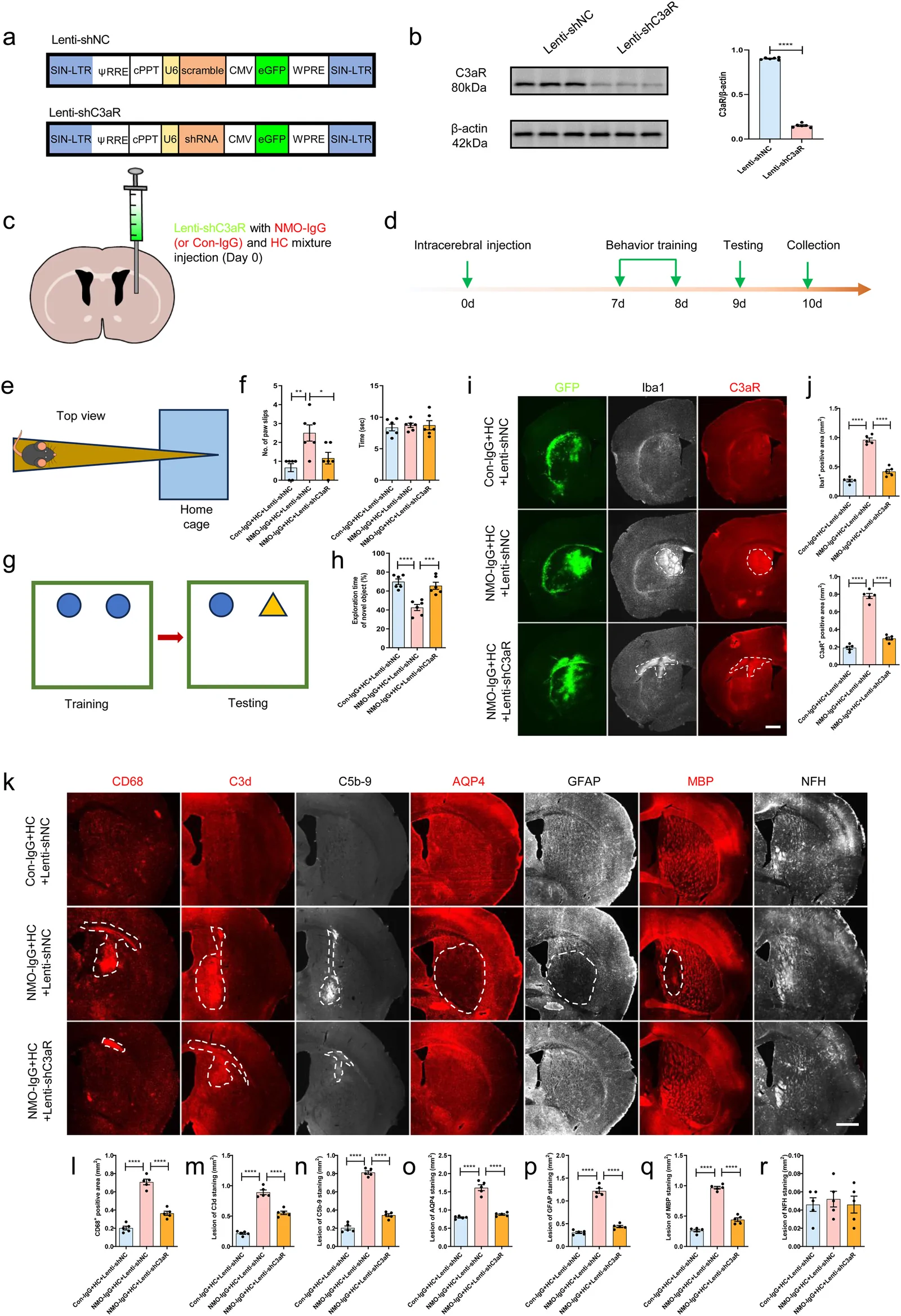
**C3aR deletion mitigates pathology and cognitive impairments in a focal intracerebral injection mouse model of NMO**. (a) Diagrams of Lenti-NC and Lenti-shC3aR constructs including SIN-LTR (self-inactivating long terminal repeat), RRE (rev response elements), CMV (cytomegalovirus) promoter, and WPRE (virus post-transcriptional regulation) elements. (b) Representative immunoblots (left) and quantification of C3aR levels (right) in HEK293T cells transduced with Lenti-NC or Lenti-shC3aR. *n* = 6 biological replicates for each group. (c, d) Schematic (c) and timeline (d) for NMO mouse model with focal intracerebral Lenti-shC3aR or Lenti-NC injections. (e) Balance beam (BB) test schematic. (f) SB-treated mice showed reduced paw slips in the BB test without increased travel time, compared to NMO mice. *n* = 6 mice for each group. (g) Novel object recognition (NOR) test schematic. (h) SB-treated mice displayed enhanced exploratory preference in the NOR test versus NMO mice. *n* = 6 mice for each group. (i) Co-immunostaining of Iba1 (silver) and C3aR (red) at the injection site with GFP (green) in corpus striatum sections; lesions outlined with dashed lines. Scale: 100 µm. (j) Quantified Iba1 and C3aR positive areas in corpus striatum sections across groups. *n* = 5 mice (3 sections per mouse) per group. (k) Immunostaining of CD68, C3d, C5b-9, AQP4, GFAP, MBP, and NFH at the injection site in corpus striatum sections; lesions outlined with dashed lines. Scale: 100 µm. (l–r) Quantified positive areas of CD68 (l), C3d (m), C5b-9 (n), and loss areas of AQP4 (o), GFAP (p), MBP (q), NFH (r) in corpus striatum sections across groups. *n* = 5 mice (3 sections/mouse) per group. Data are shown as mean ± SEM, analyzed via Student’s *t*-test for (b) and one-way ANOVA with Tukey’s post hoc test for other comparisons. *p< 0.05, **p< 0.01, ***p< 0.001, ****p< 0.0001. Non-significant results omitted.

Next, we examined the pathological changes in the corpus striatum due to C3aR knockdown. As shown in Fig. 2i, j, the area of either Iba1^+^ microglia or C3aR in Iba1^+^ microglia at the site of GFP-labelled injection was significantly decreased in mice treated with lenti-shC3aR compared with mice in the NMO group, indicating that targeted inhibition of C3aR signaling effectively reduces microglial infiltration in NMO lesions. Expectedly, C3aR-deficient mice exhibited fewer typical NMO-like lesions in the corpus striatum (Fig. 2k), including a noticeable decrease in CD68 (Fig. 2l), C3d (Fig. 2m), and C5b-9 (Fig. 2n) positive areas, as well as in AQP4 (Fig. 2o), GFAP (Fig. 2p), and MBP (Fig. 2q) negative areas, compared to NMO group mice. Likewise, NFH did not show a significant change among different groups (Fig. 2r). Collectively, these results demonstrated that blocking C3aR signaling in microglia is sufficient to limit localized NMO pathology, resulting in definitive improvement in neurological function.

### 3.3 C3aR antagonist (SB 290157 trifluoroacetate) prevents the optic nerve from inflammatory attack in the NMO eye model

Since optic neuritis, leading to impaired vision, is one of the major clinical manifestations of NMO [1], we utilized an NMO rat eye model to investigate whether C3aR antagonists had a beneficial effect on NMO-associated optic neuritis (Fig. 3a). As previously described [24], rats were subjected to visual evoked potentials (VEP) and electroretinography (ERG) before surgery, and on days 3 and 7 after sub-sheath injection of NMO-IgG (or Con-IgG) into the optic nerve, respectively (Fig. 3b). Additionally, SB group rats received an intraperitoneal (i.p.) injection of SB (20 mg/kg) every other day, starting 2 days post-operation. Compared with control group rats, both a longer average latency and a lower average amplitude of P1 were found in the VEP recordings from rats in the NMO group (Fig. 3c–e), indicating slowed optic nerve conduction velocity resulting from demyelination and axonal damage. Similarly, ERG analysis revealed a progressive reduction in PhNR amplitude under stimulation of either 3 µV or 10 µV in NMO group rats (Fig. 3f–h), suggesting an abnormal status of retinal ganglion cells (RGCs). Collectively, these findings demonstrated that rat eye models successfully mimic the visual defects of NMO. However, rats injected with SB partially avoided visual impairments, as evidenced by improved parameters in two visual electrophysiological examinations (Fig. 3c–h), which highlights the protective effect of SB in the NMO eye model.

**Fig. 3:**
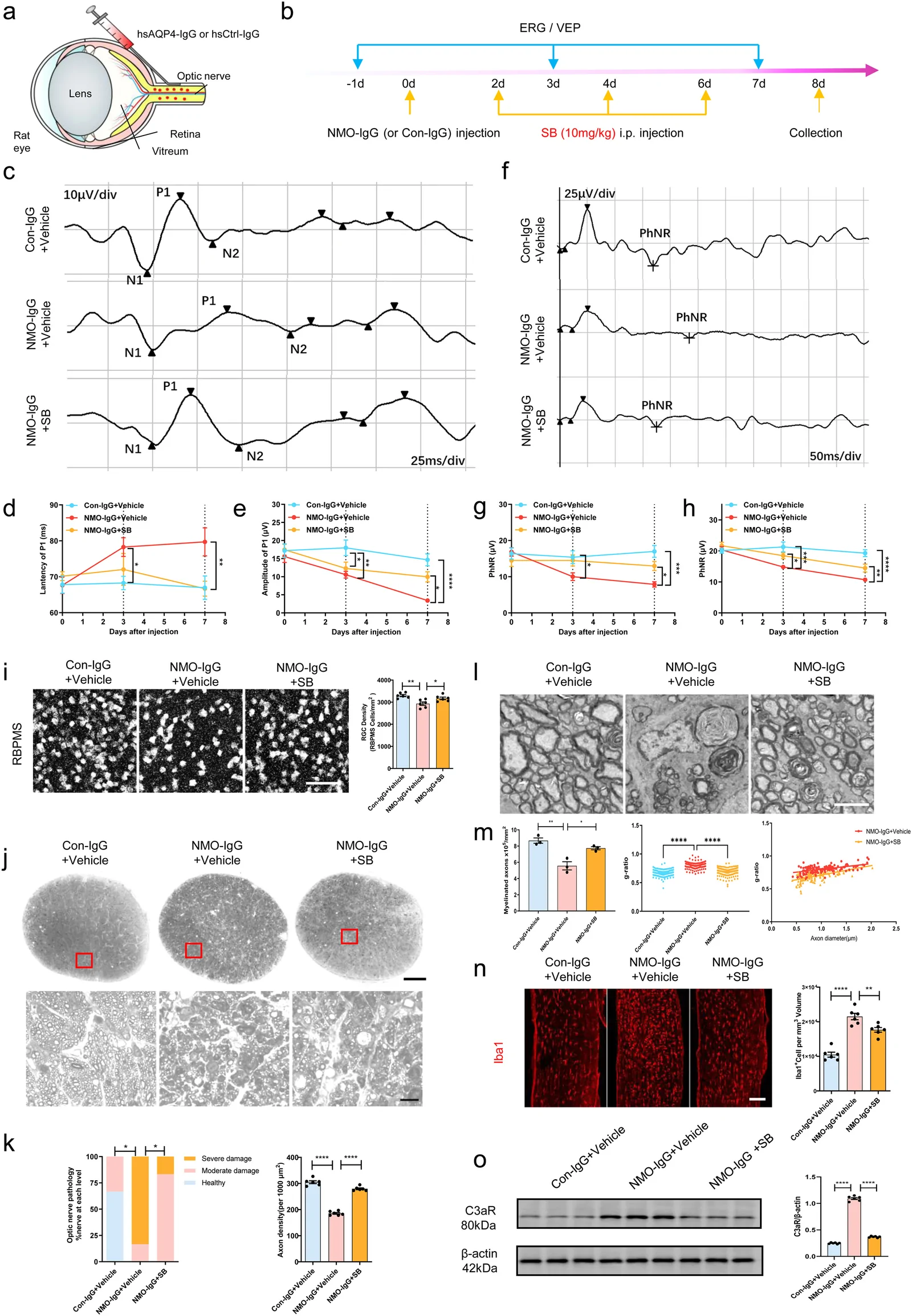
**C3aR antagonism mitigates ocular damage and visual impairment in an NMO eye model.** (a, b) Experimental design schematic (a) and timeline (b) for a rat model of NMO optic neuritis with/without C3aR antagonist (SB) treatment. (c) VEP recording waveforms 7 days post-surgery. (d, e) SB treatment resulted in reduced latency (d) and increased P1 wave amplitude (e) in VEP tests compared to NMO eyes. *n* = 6 eyes for each group. (f) ERG recording waveforms 7 days post-surgery. (g, h) SB-treated eyes showed greater PhNR amplitude with 3 µV (g) and 10 µV (h) stimuli over time than NMO eyes. *n* = 6 eyes for each group. (i) RBPMS^+^ RGCs staining and quantification in flat-mount retinas across groups. Scale: 100 µm. *n* = 6 eyes (12 sections per eye) for each group. (j) Optic nerve semithin sections of toluidine blue staining, showing insets highlighting intact axons in the SB group versus atrophied axons in the NMO group. Scale: 100 µm (upper), 20 µm (lower). (k) Optic nerve damage severity distribution and axon density in optic nerves across groups. *n* = 6 eyes (3 sections per eye) for each group. (l) Optic nerve imaging via electron microscopy. Scale: 2 µm. (m) Quantified myelinated axons (left) and G-ratio (middle) in optic nerve transections across groups. Scatterplot showing individual G-ratio values and axonal size distribution between NMO eyes and SB-treated NMO eyes. G-ratio based on 150 axons/group. *n* = 3 eyes for each group. (n) Iba1^+^ microglia staining and quantification in optic nerve longitudinal sections across groups. Scale: 200 µm. *n* = 6 eyes (3 sections per eye) for each group. (o) Representative immunoblots (left) and quantification of C3aR levels (right) in optic nerve lysates. *n* = 6 biological replicates for each group. Data (except k, left and m, right) are shown as mean ± SEM, analyzed via Kruskal-Wallis test for (k, left), scatterplot for (m, right), two-way ANOVA with Tukey’s test for d–h), and one-way ANOVA with Tukey’s test for other comparisons. *p< 0.05, **p< 0.01, ***p< 0.001, ****p< 0.0001. Non-significant results omitted.

Next, we examined the retina and optic nerve separately to comprehensively assess the C3aR blocker’s effect on ocular NMO lesions. Consistent with our previous study [24], NMO eyes showed a significant reduction in RBPMS^+^RGCs in the whole-mount retina compared to control eyes (Fig. 3i). Remarkable axonal loss in optic nerve cross-sections was also detected in NMO eyes by toluidine blue staining (Fig. 3j,k). In contrast, eyes from SB-treated rats displayed more surviving RGCs and intact axons than NMO eyes (Fig. 3i,k), indicating a partial rescue of NMO-induced damage by inhibiting C3aR signaling. Furthermore, we found that SB treatment helped NMO eyes retain more myelinated axons and have a lower G-ratio, as analyzed by electron microscopy (EM) (Fig. 3l,m). Similarly, fluorescence intensity of MBP and NFH was markedly increased in the optic nerves of SB-treated rats compared to NMO eyes (Fig. S3a,b). These findings suggested that SB benefits myelin disorganization and axonal damage during NMO progression. Unlike the cerebellar slice model and intracerebral injection mouse model, which received both NMO-IgG and HC, the rat model of NMO-associated optic neuritis was treated with NMO-IgG alone, tending to show astrocyte activation rather than astrocyte death in NMO lesions [24,32]. Indeed, we observed enhanced expression of GFAP and decreased staining levels of AQP4 in optic nerves of NMO eyes, consistent with our previous study [24], while these alterations were effectively blocked by treating rats with SB (Fig. S3c,d). This indicated that SB could prevent astrocyte injury induced by NMO-IgG. Notably, we speculated that the key to attenuating NMO-like lesions with SB therapy was to interrupt microglial C3aR activation, thereby limiting the local inflammatory response. This hypothesis was further confirmed by immunofluorescence analysis, revealing more Iba1^+^ microglia in NMO optic nerve tissue, and western blot analysis, showing higher protein levels of C3aR in optic nerve lysates from NMO rats, both compared to optic nerves of NMO rats receiving SB treatment (Fig. 3n,o). Moreover, we detected less deposition of C3d and C5b-9 in optic nerves from NMO rats with SB therapy than in those from NMO rats (Fig. S3e,f), suggesting that inhibiting the activation of the complement system may be one of the mechanisms by which SB reduces the inflammatory response. Consequently, the C3aR inhibitor could be a viable approach to alleviate pathological lesions in optic nerves as well as ameliorate visual disturbances in NMO.

### 3.4 Targeting the inhibition of C3aR signaling alleviates NMO-like lesions and motor impairment in a systemic mouse model of NMO

To explore the potential for mitigating myelitis with a C3aR antagonist, we treated a systemic NMO mouse model with SB (1 mg/kg) once a day on days 2 and 6 following NMO-IgG administration (Fig. 4a,b). This treatment aimed to explore the spinal cord lesions and locomotor dysfunction associated with NMO. Echoing our previous study [24], we first observed that mice in the NMO group exhibited a significant decrease of approximately 50 s in latency in the rotarod task (Fig. 4c) and a reduction of 1.25 cm in stride length in the gait test (Fig. 4d) on the last testing day compared with control group mice. Nevertheless, NMO mice receiving SB injections showed improvement in these motor deficits, as evidenced by their better performance in motor behavior tests (Fig. 4c,d), indicating that the C3aR blocker may be effective in ameliorating motor function decline in NMO. We then focused on changes in microglia in the spinal cord resulting from targeting the inhibition of C3aR. We found that both the number of Iba1^+^ microglia and the percentage of activated Iba1^+^ microglia (co-immunostaining with CD68) were lower in the spinal cords of NMO mice after SB administration (Fig. 4e,f). WB analysis indicated a lower expression of C3aR in the spinal cord lysates of SB-treated NMO mice than those injected with NMO-IgG alone (Fig. 4g). These observations supported the role of SB in efficiently reducing microglial activation through targeting C3aR inhibition, as previously reported [25]. Additionally, proinflammatory cytokines released by microglia, such as IL-1β, IL-6, C1q, and TNF-α, showed a significant increase, while the presence of the anti-inflammatory cytokine IL-10 was significantly decreased in the spinal cord homogenates of NMO mice (Fig. 4h). In contrast, treatment with SB in NMO mice reversed the upregulation of IL-1β, IL-6, C1q, and TNF-α and promoted the release of IL-10. These results demonstrated that SB could reduce pathological inflammation in NMO progression. A milder inflammatory response theoretically contributes to the mitigation of lesions. Following this notion, we assessed the severity of NMO-like lesions in the spinal cord among different groups of mice. Compared to NMO mice treated with SB, spinal cords from mice infused with NMO-IgG showed a significant reduction in the number of NeuN^+^ neurons, particularly in the ventral horn, which is closely related to limb activity (Fig. 4i). The fluorescence signals of AQP4, MBP, and NFH in transverse sections of the spinal cord in the NMO group mice also decreased dramatically relative to those in the SB treatment group (Fig. 4j–l). In summary, pharmacological inhibition of C3aR with SB might be a therapeutic intervention for NMO attacks.

**Fig. 4:**
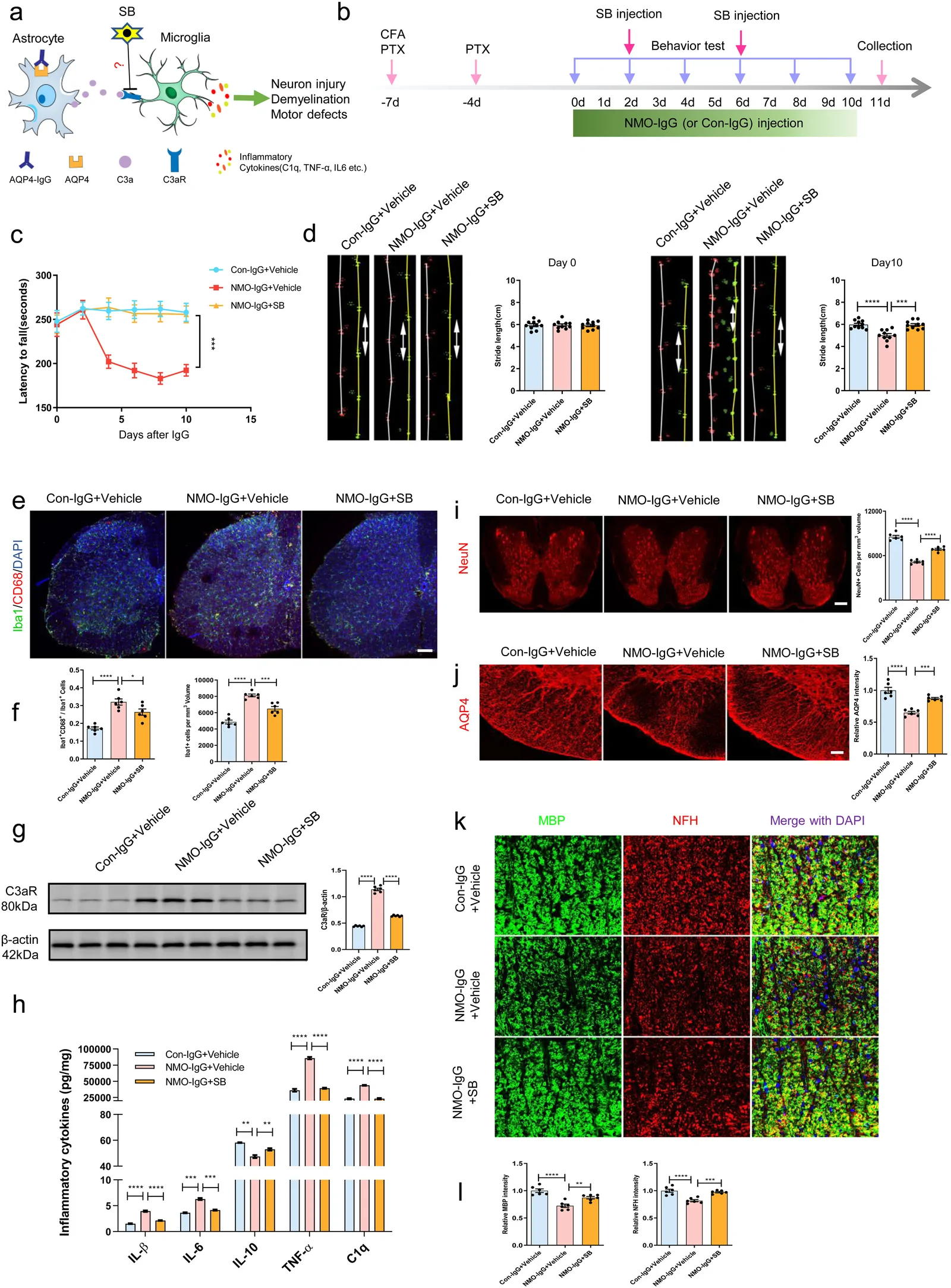
**C3aR antagonist reduces locomotor deficits and microglial inflammation in systemic NMO model**. (a) Diagram illustrating C3aR antagonist (SB) blocking C3-C3aR interaction to prevent NMO-like lesions. (b) Timeline for systemic NMO mouse model experimental design with/without SB treatment. (c) SB-treated mice showed increased latency to fall in the rotarod test compared to NMO mice. *n* = 10 mice for each group. (d) Gait test images and stride length analysis show longer strides in SB-treated mice on day 10 versus NMO mice. *n* = 10 mice for each group. (e) Iba1 (green) and CD68 (red) co-staining with DAPI (blue) in spinal cord sections across groups. Scale: 200 µm. (f) Iba1^+^ microglia count and CD68^+^ Iba1^+^ microglia percentage in spinal cord sections across groups. *n* = 6 mice (3 sections per mouse) for each group. (g) Representative immunoblots (left) and quantification of C3aR levels (right) in spinal cord lysates across groups. *n* = 6 biological replicates for each group. (h) Inflammatory cytokines (IL-1β, IL-6, IL-10, TNF-α, C1q) quantification in spinal cord homogenates across groups. *n* = 3 biological replicates for each group. (i) NeuN^+^ neuronal cell staining and quantification in spinal cord sections across groups. Scale: 400 µm. *n* = 6 mice (3 sections per mouse) for each group. (j) AQP4 staining and relative immunofluorescence intensity in spinal cord sections across groups. Scale: 100 µm. *n* = 6 mice (3 sections per mouse) for each group. (k) MBP and NFH co-staining in spinal cord white matter across groups. Scale: 100 µm. (l) MBP and NFH relative immunofluorescence intensity in spinal cord white matter across groups. *n* = 6 mice (3 sections per mouse) for each group. Data are shown as mean ± SEM, analyzed via two-way ANOVA with Tukey’s test for (c) and one-way ANOVA with Tukey’s test for other comparisons. *p< 0.05, **p< 0.01, ***p< 0.001, ****p< 0.0001. Non-significant results omitted.

### 3.5 Phosphorylation of STAT3 signaling through C3-C3aR activation induced by NMO-IgG in vitro and *ex vivo* models

A recent study has identified that STAT3 may be a specific target of C3aR, based on findings that only STAT3 expression was rescued by C3aR knockout (KO) in bulk mouse brain samples and in purified microglia [8]. To verify whether STAT3 activation also occurs in microglia in NMO (Fig. 5a), we first treated BV2 cells with C3 protein and observed an induced surge in the translocation of STAT3 phosphorylation (p-STAT3) to the nucleus of BV2 cells compared to PBS-treated cells (Fig. 5b). This result was further confirmed by WB analysis of cell lysates, showing markedly elevated p-STAT3 protein levels with C3 supplementation (Fig. 5c), suggesting that STAT3 can be activated by C3-C3aR in BV2 microglia. This supports the hypothesis that C3aR may function as the upstream mediator of STAT3 activation. However, the appearance of nuclear p-STAT3 staining significantly decreased in BV2 microglia treated with the culture medium from astrocytes stimulated by Lenti-shC3 and NMO-IgG (Fig. S4a,b). Together, these findings suggest that activated C3-C3aR signaling by NMO-IgG provides modulation of p-STAT3 activity in microglia.

**Fig. 5:**
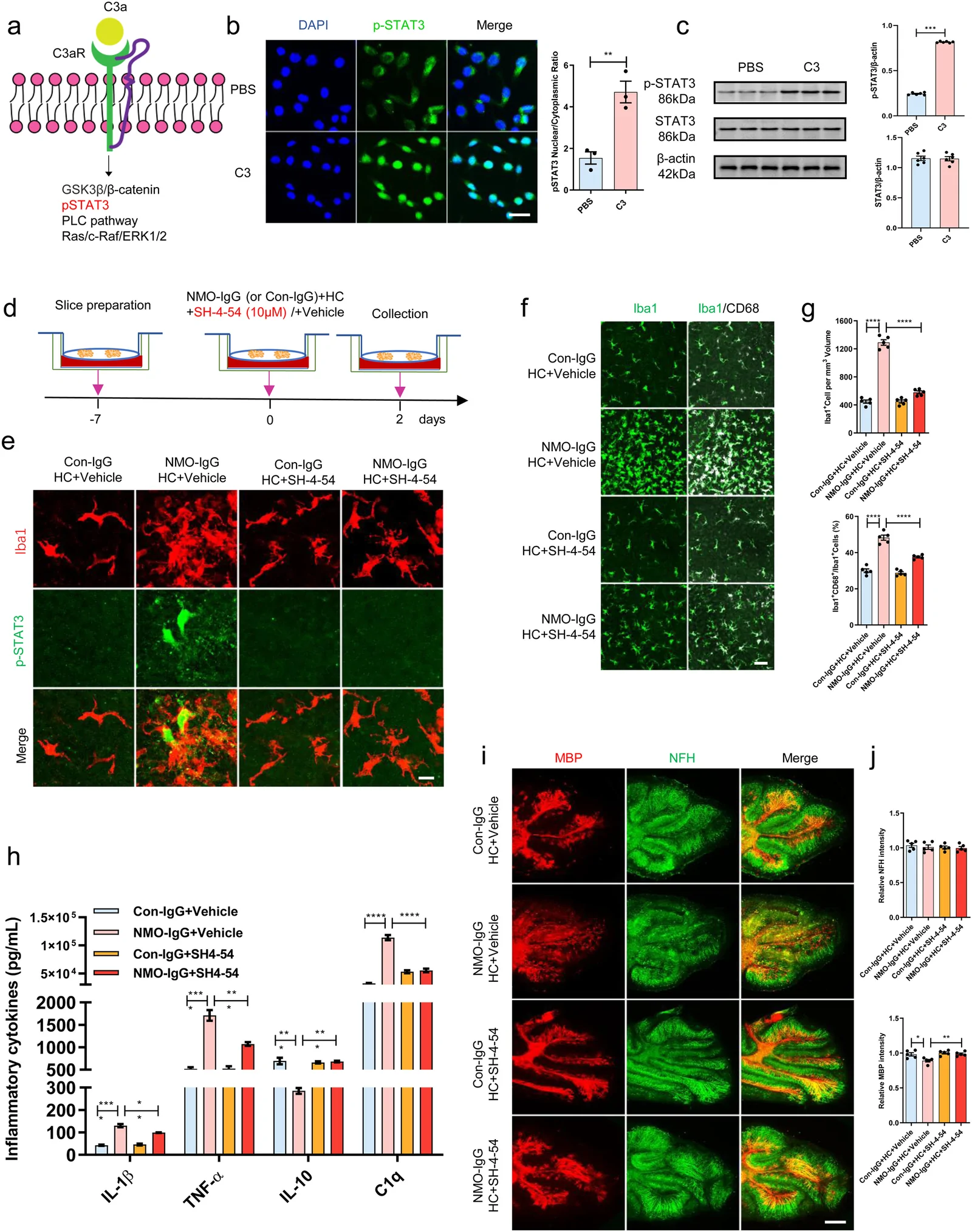
**C3aR** s**ignaling** r**egulates STAT3 phosphorylation in microglia during NMO**. (a) Diagram outlining potential downstream targets of C3-C3aR signaling. (b) p-STAT3 (green) and DAPI (blue) co-staining in BV2 cells treated with PBS or human C3 protein for 24h Scale: 20 µm. *n* = 3 slides for each group (6 sections per slide, 50 cells per section). (c) Representative immunoblots (left) and quantification of pSTAT3/β-actin (right upper) and STAT3/β-actin (right lower) levels in treated BV2 cells across groups. *n* = 6 biological replicates. (d) Schematic and timeline for NMO cerebellar slice model with SH-4-54 or DMSO treatment. (e) p-STAT3 (green) and Iba1 (red) co-staining in cerebellar slices across groups. Scale: 50 µm. (f) Iba1 (green) and CD68 (silver) co-staining in cerebellar slices across groups. Scale: 100 µm. (g) Iba1^+^ microglia count and CD68^+^ Iba1^+^ microglia percentage in cerebellar slices across groups. *n* = 5 slices (3 sections per slice) for each group. (h) Inflammatory cytokines (IL-1β, IL-10, TNF-α and C1q) quantification in slice culture medium across groups. *n* = 3 biological replicates for each group. (i) MBP (red) and NFH (green) co-staining in cerebellar slices across groups. Scale: 100 µm. (j) MBP and NFH relative immunofluorescence intensity in cerebellar slices across groups. *n* = 5 slices (3 sections per slice) for each group. Data are shown as mean ± SEM, analyzed via Student’s *t*-test for (b and c) and one-way ANOVA with Tukey’s post hoc test for other comparisons. *p< 0.05, **p< 0.01, ***p< 0.001, ****p< 0.0001. Non-significant results omitted.

Extending this, blocking p-STAT3 might confer similar efficacy to C3aR inactivation. To test this hypothesis, we used a commercially available STAT3 phosphorylation site inhibitor, SH-4-54, first in *ex vivo* models of NMO (Fig. 5d). As expected, SH-4-54 effectively blocked STAT3 phosphorylation in microglia of cerebellar slices treated with NMO-IgG and HC (Fig. 5e), which resulted in a significant reduction both in the total number of Iba1^+^ microglia and in the percentage of CD68^+^ Iba1^+^ activated microglia (Fig. 5f,g). Additionally, the contents of proinflammatory cytokines (IL-1β, C1q, and TNF-α) released by microglia saw a visible decline, while the protein levels of the anti-inflammatory cytokine IL-10 apparently increased in the medium of NMO slices with SH-4-54 compared to NMO group slices (Fig. 5h). These observations imply that inhibition of p-STAT3 could limit microglial aggregation and activation to mediate inflammatory overreaction in NMO.

Immunostaining analysis using antibodies against MBP and NFH demonstrated significant protection of myelin and axons in slices treated with NMO-IgG and SH-4-54 compared to those in the NMO group (Fig. 5i,j). This result correlated with higher fluorescence intensity of AQP4 and GFAP, as well as a lower NMO-like lesions score in the SH-4-54 treatment group slices compared to those in the NMO group (Fig. S4c,d). Moreover, fewer depositions of C3d and C5b-9 were also found in NMO slices with SH-4-54 treatment (Fig. S4e). Taken together, these findings suggest that SH-4-54 is effective in mitigating NMO-like lesions through inhibiting the phosphorylation of STAT3 in microglia.

### 3.6 Pharmacological inhibition of p-STAT3 (SH-4-54) improves locomotor and visual dysfunctions in NMO experimental models

Considering the positive role of SH-4-54 on NMO pathology, we assessed its benefits for neurological deficits in NMO. Similar to the use of a C3aR antagonist, rats with optic neuritis related to NMO were administered SH-4-54 via i.p. injection every other day starting on day 2 after NMO-IgG stimulation (Fig. 6a). As expected, SH-4-54 treatment allowed rats injected with NMO-IgG to maintain both the latency and amplitude of the P1 wave almost equal to those in the control group rats in the VEP examination (Fig. 6b,c). In line with the VEP test, it significantly increased the amplitude of PhNR in rats receiving NMO-IgG with a test condition of 3 µV or 10 µV in the ERG project (Fig. 6d,e). These electrophysiology assessments implicated a potent role of p-STAT3 blockade in rescuing visual disability in NMO. We then explored the pathological changes in the optic nerves among different groups of rats. Similarly, more Iba1^+^ microglia with higher expression of p-STAT3 were detected in optic nerves of NMO group rats (Fig. 6f), which led to weaker staining signals of MBP (Fig. 6g,h upper), NFH (Fig. 6g,h lower), and AQP4 (Fig. S5a) while showing stronger fluorescence intensity of GFAP (Fig. S5b), C3d (Fig. S5c), and C5b-9 (Fig. S5d) than those in optic nerves from SH-4-54-treated rats. This supports the notion that inhibition of p-STAT3 in microglia could restrict microgliosis and dampen neuroinflammation in NMO-like lesions. Moreover, SH-4-54 treatment protected more RGCs and axons of optic nerves from the inflammatory attack induced by NMO-IgG compared to the NMO group rats (Fig. 6i,j).

**Fig. 6:**
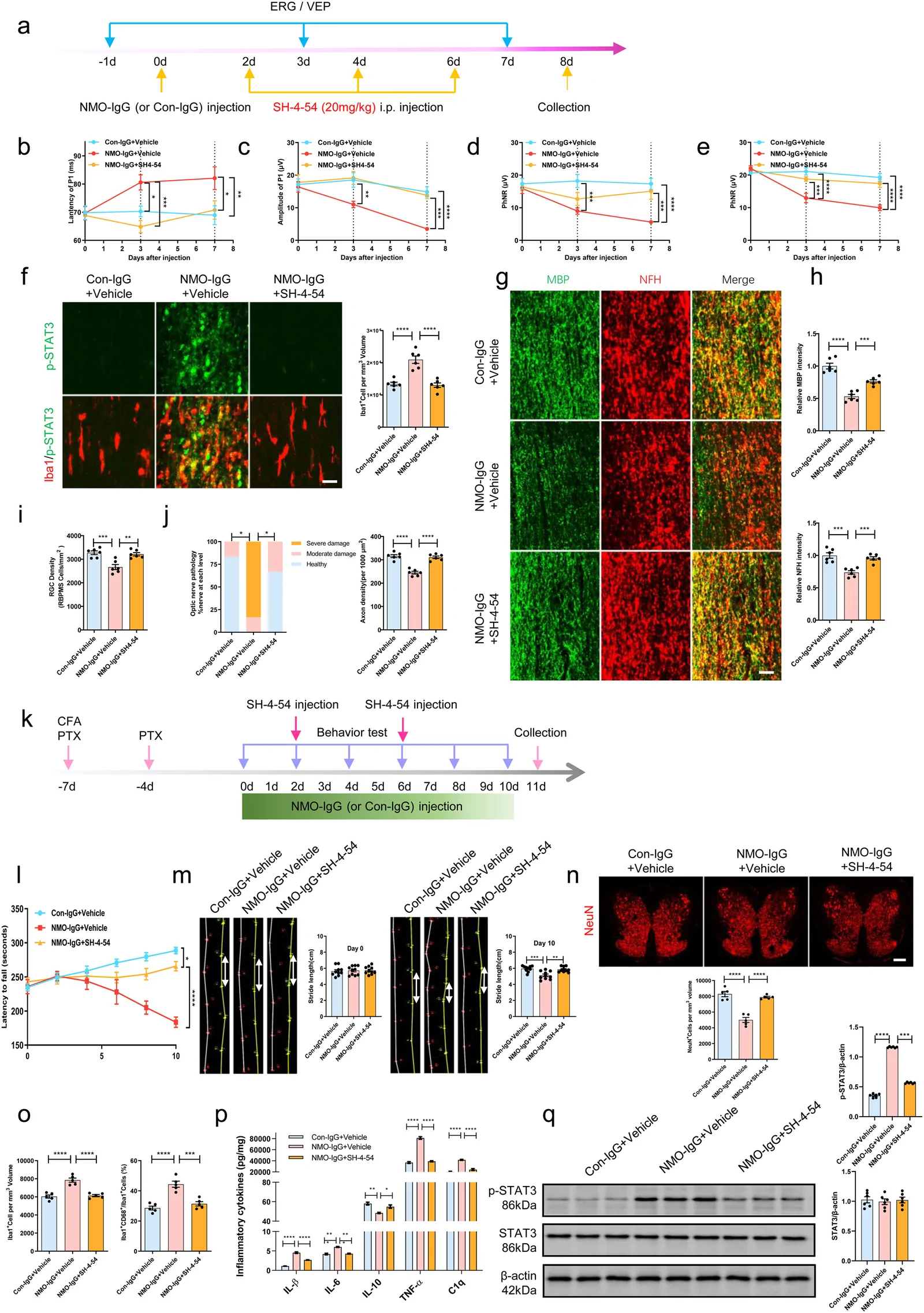
**SH-4-54 enhances recovery in NMO models by targeting p-STAT3.** (a) Experimental timeline for a rat NMO optic neuritis model with/without SH-4-54 treatment. (b, c) SH-4-54 treatment improved P1 wave latency (b) and amplitude (c) in VEP tests compared to NMO rats. *n* = 6 eyes for each group. (d, e) SH-4-54-treated eyes exhibited increased PhNR amplitude with 3 µV (d) and 10 µV (e) stimuli over time versus NMO eyes. *n* = 6 eyes for each group. (f) p-STAT3 and Iba1 co-staining in optic nerves, showing Iba1^+^ microglia count across groups. Scale: 50 µm. *n* = 6 eyes (3 sections per eye) for each group. (g) MBP and NFH co-staining in the optic nerve across groups. Scale: 100 µm. (h) MBP and NFH relative immunofluorescence intensity in the optic nerve across groups. *n* = 6 eyes (3 sections per eye) for each group. (i) RBPMS^+^RGCs count in flat-mount retinas across groups. *n* = 6 eyes (12 sections per eye) for each group. (j) Severity distribution and axon density in the optic nerve across groups. *n* = 6 eyes (3 sections per eye) for each group. (k) Timeline for systemic NMO mouse model with/without SH-4-54 treatment. (l) SH-4-54-treated mice showed improved rotarod test performance versus NMO mice. *n* = 10 mice for each group. (m) Gait analysis showing longer stride length in SH-4-54-treated mice on day 10 compared to NMO mice. *n* = 10 mice for each group. (n) NeuN^+^ neuronal cell staining and quantification in spinal cord sections across groups. Scale: 400 µm. *n* = 5 mice (3 sections per mouse) for each group. (o) Iba1^+^ microglia count and CD68^+^ Iba1^+^ microglia percentage in spinal cord sections across groups. *n* = 5 mice (3 sections per mouse) for each group. (p) Inflammatory cytokine (IL-1β, IL-6, IL-10, TNF-α and C1q) quantification in spinal cord homogenates across groups assessed by ELISA. *n* = 3 biological replicates for each group. (q) Representative immunoblots (left) and quantification of pSTAT3/β-actin (right upper) and STAT3/β-actin (right lower) levels in spinal cord lysates across groups. *n* = 6 biological replicates for each group. Data (except j, left) are shown as mean ± SEM, analyzed via two-way ANOVA with Tukey’s test for (b–e, l), Kruskal-Wallis for (j, left), and one-way ANOVA with Tukey’s test for other comparisons. *p< 0.05, **p< 0.01, ***p< 0.001, ****p< 0.0001. Non-significant results omitted.

Similarly, we explored the effect of SH-4-54 on motor dysfunction in NMO using a systemic mouse model (Fig. 6k). As anticipated, NMO group mice performed worse in two motor behavior tasks than the control group and SH-4-54 group mice (Fig. 6l,m), implying that the p-STAT3 inhibitor could reverse locomotor impairments associated with NMO in vivo. Given that functional improvements imply recovery from localized lesions, we analyzed the spinal cord from different groups of mice. Immunofluorescent staining revealed more surviving NeuN^+^ motor neurons in the ventral horn of the spinal cord from SH-4-54-treated mice than from the NMO group mice (Fig. 6n), suggesting that SH-4-54 might favor neuron survival by preventing inflammatory damage during NMO progression. This deduction was further validated by a marked decline in the number of Iba1^+^ microglia and the percentage of CD68^+^ proinflammatory microglia in the spinal cord of SH-4-54 treatment group mice compared with NMO group mice (Fig. 6o), accompanied by decreased secretion of IL-1β, IL-6, C1q, and TNF-α, and an increased content of IL-10 in spinal cord homogenates (Fig. 6p). Importantly, lower expression only of p-STAT3, not of total STAT3, was identified in the spinal cord lysates of mice administered NMO-IgG and SH-4-54 than in the NMO group mice (Fig. 6q), inferring that blocking the activation of STAT3 in microglia is a crucial step in reducing inflammation. Similar to the protective role of C3aR inhibitors on NMO pathology, suppressing NMO-IgG-induced STAT3 phosphorylation downstream of C3aR effectively alleviated astrocyte injury and demyelination in the local lesion, as manifested by higher fluorescence intensity of AQP4 (Fig. S5e,f left), GFAP (Fig. S5e,f right), MBP (Fig. S5g,h upper), and NFH (Fig. S5g,h lower) in the spinal cord of SH-4-54 group mice compared to NMO group mice. Collectively, these findings conclude that pharmacological inhibition of p-STAT3 in microglia is an effective strategy for diminishing neuroinflammation, attenuating NMO pathology, and reversing locomotor and visual dysfunctions in NMO.

## 4 Discussion

Emerging evidence has supported the interaction between astrocytes and microglia contributing to neuroinflammation in NMO [11]. In our previous study, we demonstrated that NMO-IgG–induced astrocytic C3 production, accompanied by microglial activation, and that blocking C3 activation could significantly reduce microgliosis in NMO pathology [24]. In this study, we further confirmed that microglia are activated by astrocytic C3 binding to C3aR on their surface in vitro. We then identified that targeting the inhibition of C3aR leads to the attenuation of neuroinflammation, alleviation of typical pathology, and amelioration of neurological impairments in different experimental models of NMO, both *ex vivo* and in vivo. Moreover, we revealed that microglial p-STAT3 is a downstream target of C3-C3aR signaling induced by NMO-IgG. We documented that a p-STAT3 blocker is sufficient to correct local pathogenic lesions as well as rescue visual and motor deficits. In conclusion, our study reveals the mechanism of the microglial C3aR-pSTAT3 axis in NMO pathogenesis and provides novel insights into investigating this pathway for therapeutic intervention.

Microglial activation is generally accepted as being crucial for neuroinflammatory damage during an NMO attack [12]. A recent study reported that NMO-IgG stimulation increases the crosstalk between astrocytes and microglia through C3-C3aR signaling in an NMO mouse model with motor impairments [11]. Consistent with this [11,33], we verified that the expression of C3aR on BV2 microglia remarkably increased under the condition of adding either human C3 protein or glial conditioned medium treated with NMO-IgG, which has been demonstrated to result in a significant increase in C3 production from astrocytes [24]. These findings support that C3aR on microglia is upregulated in NMO. In fact, numerous studies have found that enhanced C3aR signaling in microglia exacerbates inflammation-induced cognitive impairment in AD [25], hydrocephalus of germinal matrix hemorrhage and intraventricular hemorrhage (GMH-IVH) [26], white matter injury of hypoperfusion [30], and other immunoregulation and neuroinflammatory diseases [34,35], suggesting that C3aR upregulation in microglia is associated with inflammatory overaction. As native macrophages in the CNS, microglia are the major mediators of CNS inflammation, mainly through releasing pro- and anti-inflammatory cytokines [36]. Consistent with other studies [8,11], we observed that C3aR upregulation promotes activated microglia to amplify the inflammatory response, evidenced by secreting more proinflammatory factors (C1q, IL-1β, and TNF-α) and fewer anti-inflammatory factors (IL-10) in the culture medium after binding to C3. Among these cytokines, C1q not only activates the classic complement cascade, bringing about an inflammatory attack [12], but also directly induces axonal damage and neuronal stress [14,19]. As for IL-1β and TNF-α, both are involved in the pathological process of NMO [37], leading us to conclude that C3aR might be a potential target in microglia to regulate the inflammation response in NMO.

Furthermore, the expression of C3aR on microglia was also found to be markedly increased in *ex vivo* and in vivo models of NMO by WB and immunofluorescence analysis, coinciding with AQP4 loss, astrocyte injury, myelinolysis, neuronal damage, and complement deposition, indicating that local tissues suffer pronounced inflammatory attacks. This notion was further verified by detecting microglia activation in the same position as NMO-like lesions, as well as proinflammatory cytokine secretion in NMO models of the supernatant of organotypic cerebellar slice and spinal cord homogenates. These pathological changes related to C3aR overexpression on microglia are in line with a pronounced inflammatory response seen in AD mice and GMH-IVH rats [8,25,26]. Recently, a study demonstrated that genetic deletion of C3aR in microglia mitigates NMO pathology in the spinal cord as well as motor impairment [11]. Considering gene deletion is difficult to apply in human NMO, we used SB, a highly selective C3aR nonpeptide antagonist widely tested in multiple studies, to block C3aR signaling in microglia across various experimental models of NMO. Surprisingly, we found that SB treatment provides benefits beyond reducing neuron loss and improving limb disability, similar to those seen in C3aR KO mice [11]. It plays an active role in lessening NMO-like lesions of the target brain and optic nerve tissues as well as ameliorating balance disorder, cognitive decline, and visual impairment in corresponding models of NMO. The therapeutic effects of SB in NMO may be attributed to reduced inflammation by blocking astrocytic C3 binding to C3aR on microglia, preventing the proliferation and activation of resting microglia, resulting in fewer transformations to the M1 proinflammatory microglia type. Similar to targeting the inhibition of C3 [24], SB’s effect on disrupting C3aR signaling could reduce the excess accumulation of proinflammatory factors by microglia in NMO models, effectively halting microglia-associated proinflammatory activity, as demonstrated in various immune-inflammatory diseases [8,11,25,26,30]. These findings support that pharmacological inhibition of C3aR with SB treatment is a viable therapeutic intervention in NMO.

Previous studies have shown that microglia can be activated by IL-6 and type I interferons (IFN-Is) during NMOSD, leading to STAT activation [13,38,39]. Researchers recently found that STAT3 is a target of C3-C3aR signaling that functionally mediates tau pathogenesis in AD mice [8]. Accordingly, we speculated that microglia activated by C3aR binding to C3 may also undergo phosphorylation of STAT3 signaling. This hypothesis was verified in vitro, *ex vivo*, and in vivo models of NMO, showing enhanced expression of p-STAT3 in microglia. Activated STAT3 may translocate into the nuclei of microglia and ultimately upregulate the gene expression of iNOS, IL-1α, IL-1β, and TNF-α [13], leading to inflammatory overreaction. In accord with promoting the formation and deposition of tau protein in AD mice [8], we identified that higher p-STAT3 signals in microglia in response to C3 facilitate localized injury to oligodendrocytes and neurons, resulting in visual and motor impairments. However, we revealed that the phosphorylation inhibitor SH-4-54 reduced neuroinflammation and partially rescued neurological defects in NMO. This suggests that a direct interaction of the C3aR-STAT3 axis in microglia contributes to the pathogenesis of NMO. Interestingly, inhibiting the activation of STAT3 plays a more protective role than a C3aR blocker in the experimental animal models of NMO. This could be interpreted as more than one pathway in NMO capable of activating STAT3. Typical STAT3 signaling consists of cytokine-induced, Janus kinase (JAK)-dependent STAT3 phosphorylation and nuclear translocation [40]. Besides STAT3 activation being directly regulated by C3aR in microglia [8], it has been reported that NMO-IgG can stimulate the astrocytic JAK1/2/STAT3-dependent inflammatory response through the IL-6 cascade [41]. Therefore, SH-4-54 treatment is inferred to be effective in inhibiting inflammation arising from both microglia and astrocytes, rather than only one cell type in NMO. Notably, some studies have indicated that activated microglia induce A1 astrocytes by secreting IL-1α, TNF-α and C1q, and that these cytokines together are necessary and sufficient to induce A1 astrocytes [[6], [7]], which means SH-4-54 (as a STAT3 phosphorylation site inhibitor) may effectively alleviate inflammatory damage by inhibiting the activation of astrocytes, through mechanisms such as preventing activated microglia from secreting pro-inflammatory cytokines or inhibiting the self-activation of astrocytes. In fact, an independent study documented a role for the IL-10-STAT3 pathway in mediating microglial Ab phagocytosis recently [42]. This reminds us that other possible mechanisms of STAT3 activation in NMO remain to be investigated.

## 5 Conclusion

In sum, this study identified the pathogenic role of microglial C3aR in local inflammatory lesions as well as neurological dysfunctions in vitro, e*x vivo*, and in vivo models of NMO. Importantly, we first revealed the mechanism by which the crosstalk between STAT3 and C3aR in microglia mediates neuroinflammation in NMO. Pharmacological intervention of C3aR or p-STAT3 effectively attenuates inflammatory damage in target CNS tissue, resulting in significant improvements in vision loss and limb weakness during NMO progression. Additionally, using LVs to knock down the expression of C3aR in the corpus striatum also mitigates NMO-like lesions and partially rescues dystaxia and dysmnesia related to NMO. Hence, this study unveils a novel mechanism of C3aR-STAT3 signaling in microglia that contributes to NMO pathology and offers potential therapeutic options for this pathway in NMO and other autoimmune inflammatory diseases.

## CRediT authorship contribution statement

**Li Xu:** Writing – original draft, Validation, Software, Methodology, Formal analysis, Data curation. **Xinyi Wang:** Methodology, Data curation. **Huiming Xu:** Software, Methodology, Data curation. **Siqi Chen:** Software, Methodology, Data curation. **Wei Jiang:** Methodology, Funding acquisition. **Haoyang Li:** Software, Methodology, Data curation. **Shabbir Khan Afridi:** Writing – review & editing. **Yuge Wang:** Resources. **Wenjing Luo:** Supervision, Resources, Funding acquisition. **Hui Yang:** Visualization, Resources. **Changyong Tang:** Supervision, Funding acquisition, Conceptualization. **Wei Qiu:** Supervision, Project administration, Conceptualization.

## Declaration of competing interest

The authors declare that they have no conflicts of interest in this work.

## Appendix Supplementary materials

Image, application 1

Image, application 2
