# Supplementary material for: Targeting C3aR/STAT3 signaling attenuates neuroinflammation and reverses neurological impairment in Neuromyelitis optica
Source: Fundam Res. 2025 Nov 7;6(5):3491–506. doi: 10.1016/j.fmre.2025.11.001 (PMC13644504; doi:10.1016/j.fmre.2025.11.001)
Supplement: Supplementary file 1 [file mmc1.pdf]

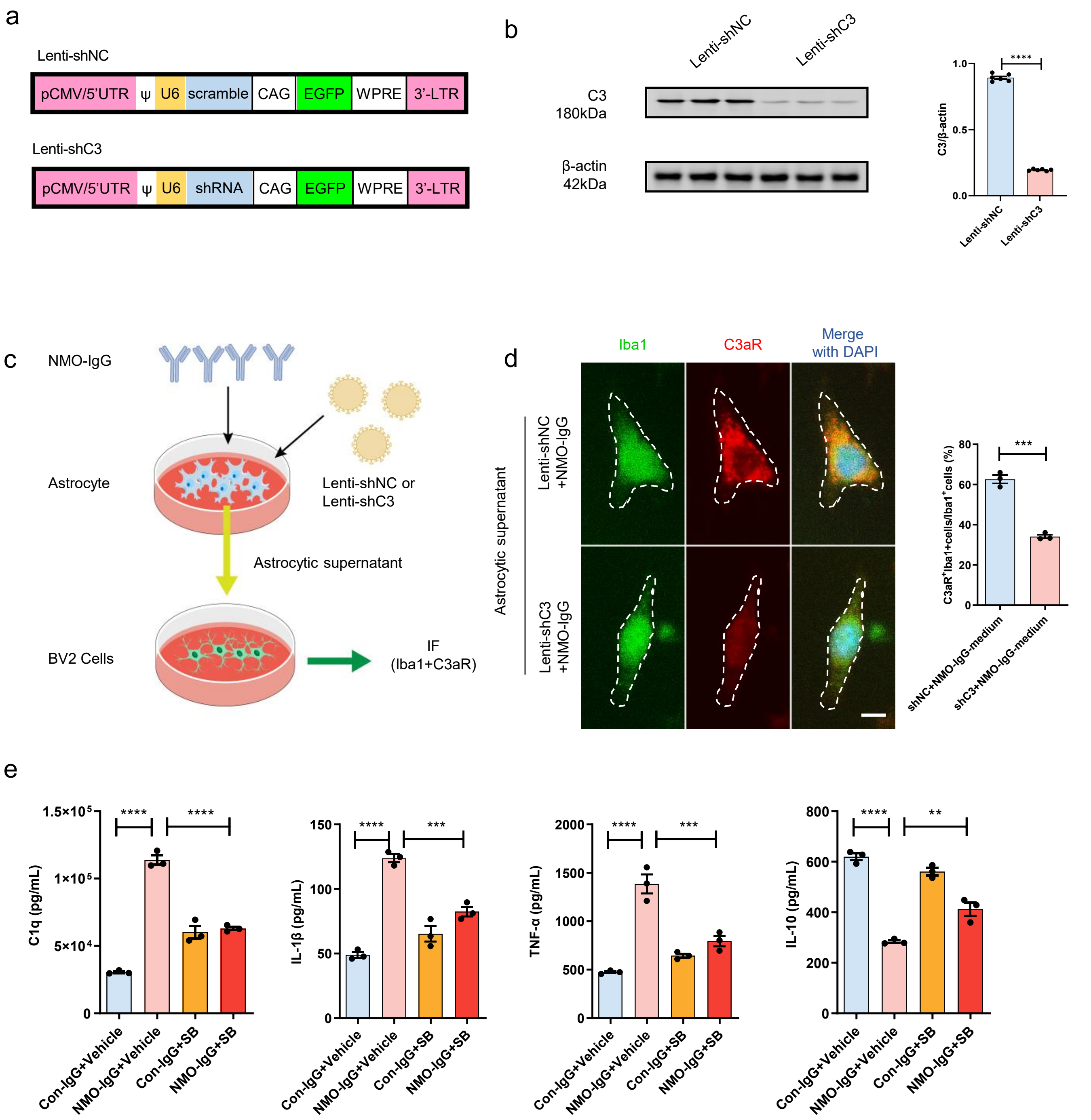

Figure S1. Regulation of C3aR in microglia by astrocytic C3 contributes to neuroinflammation upon NMO-IgG stimulation

(a) Schematic of Lenti-NC and Lenti-shC3 vectors showing key elements: CMV (cytomegalovirus promoter), untranslated region (UTR), WPRE (woodchuck hepatitis virus post-transcriptional regulatory element), and LTR (long terminal repeat).

(b) Representative immunoblots (left) and quantification of C3 levels (right) in cell lysates from Lenti-NC and Lenti-shC3 transduced primary astrocytes. n=6 biological replicates/group.

(c) Diagram illustrating the in vitro cell assay setup.

(d) Iba1 (green) and C3aR (red) co-staining with DAPI (blue), showing C3aR⁺ Iba1⁺ microglia percentage in BV2 cells exposed to various astrocytic culture mediums for 24 hours. Scale: 20 μm. n=3 slides (6 sections per slide) for each group.

(e) Inflammatory cytokines (IL-1β, IL-10, TNF-α, and C1q) quantification in slice culture medium across groups. n=3 biological replicates for each group.

Data are shown as mean ± SEM, analysed via Student's t-test for (b, d), and one-way ANOVA with Tukey's post hoc test for other comparisons. *$p < 0.05$, **$p < 0.01$, ***$p < 0.001$, ****$p < 0.0001$*. Non-significant results omitted.

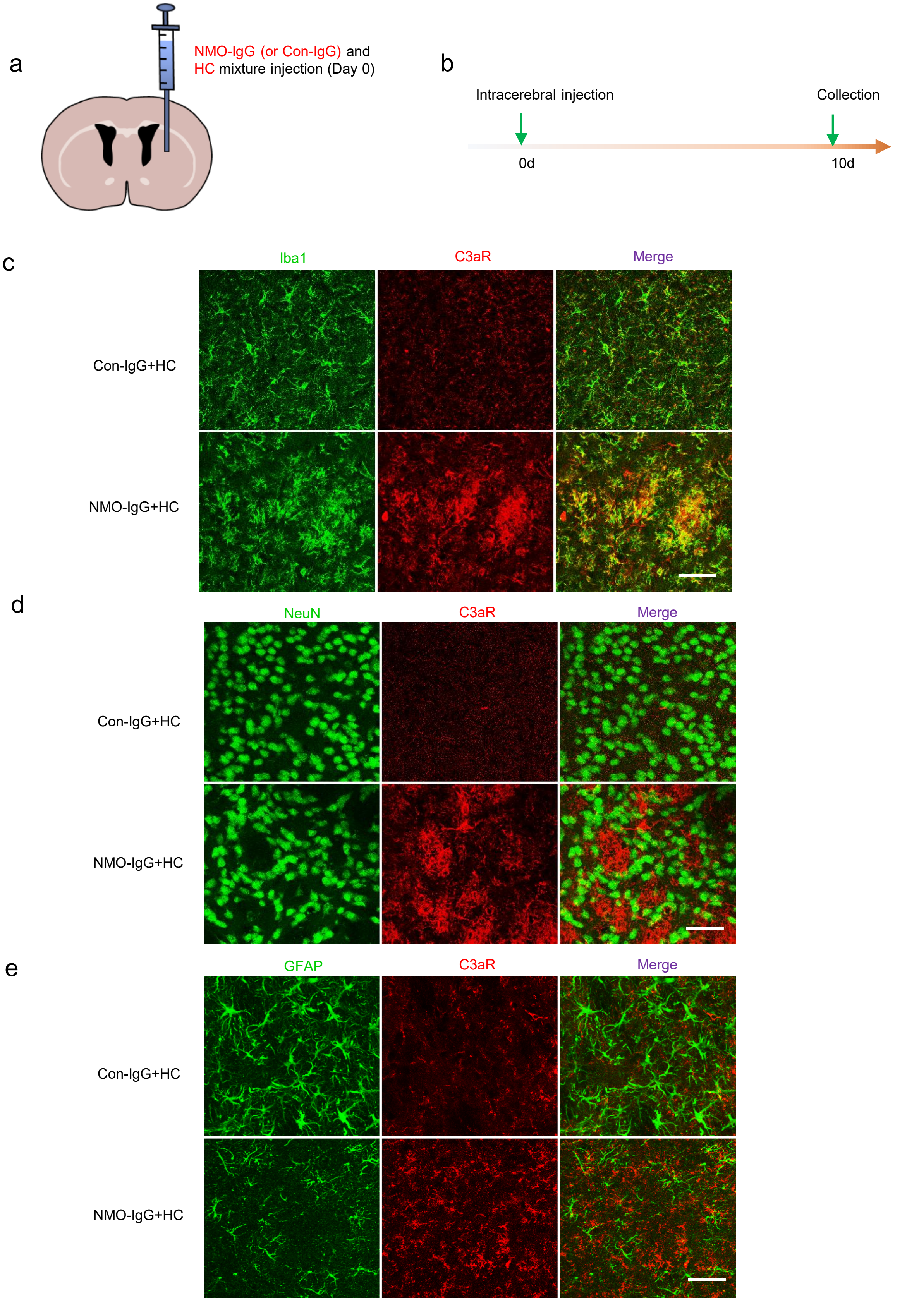

Figure S2. C3aR was mainly expressed on microglia in the corpus striatum of a focal intracerebral injection mouse model of NMO.
(a and b) Schematic (a) and timeline (b) for a focal intracerebral injection mouse model of NMO.
(c) Iba1 (green) and C3aR (red) co-staining in the corpus striatum of mice between two groups. Scale: 200 μm.
(d) NeuN (green) and C3aR (red) co-staining in the corpus striatum of mice between two groups. Scale: 200 μm.
(e) NeuN (green) and C3aR (red) co-staining in the corpus striatum of mice between two groups. Scale: 200 μm.

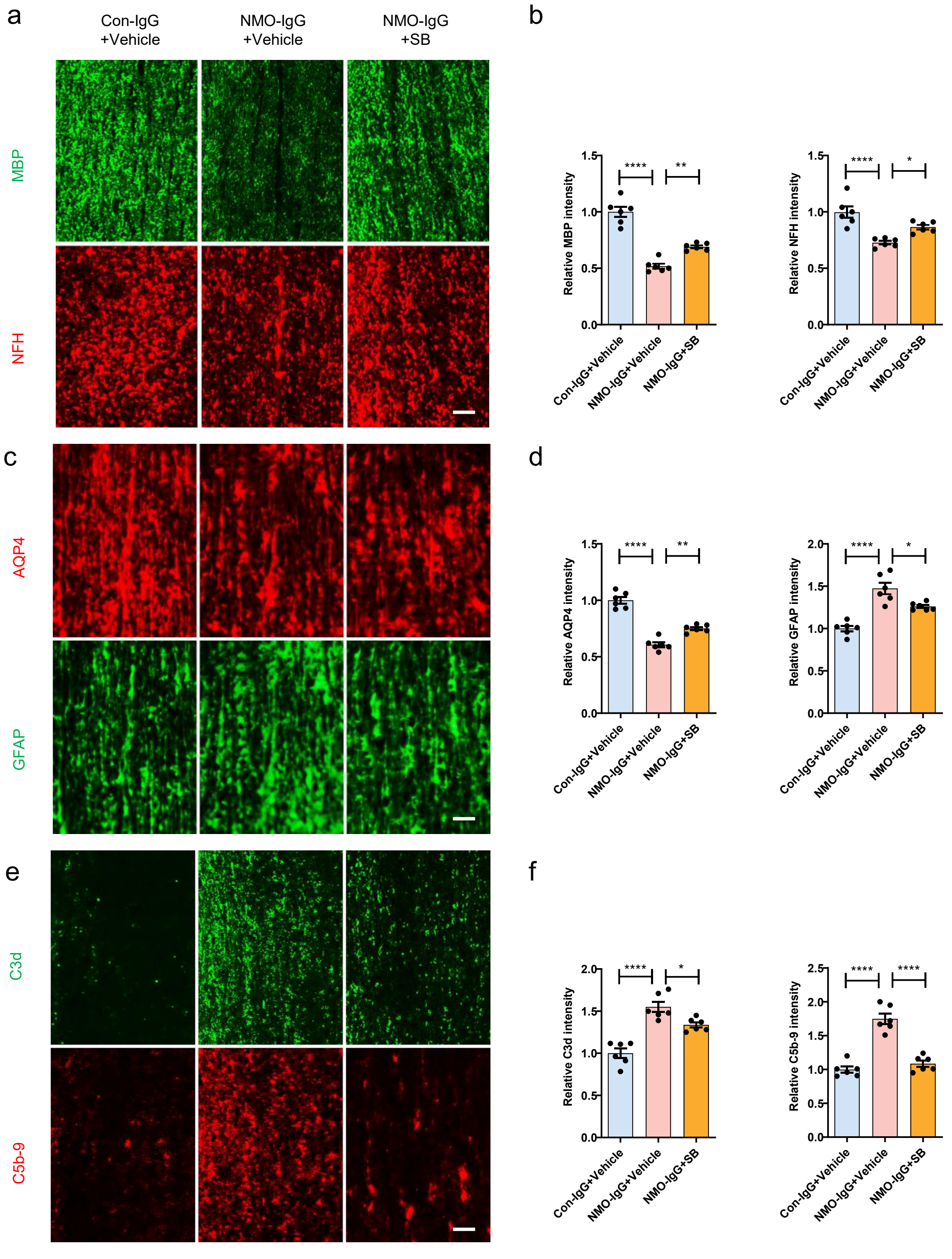

Figure S3. C3aR antagonist mitigates myelin loss, astrocyte damage, and complement deposition in an NMO-related optic neuritis rat model

(a) MBP (green) and NFH (red) co-staining in optic nerves across groups. Scale: 100 μm.

(b) MBP (left) and NFH (right) relative immunofluorescence intensity in optic nerves across groups. n=6 eyes (3 sections per eye) for each group.

(c) AQP4 (red) and GFAP (green) staining in optic nerves across groups. Scale: 100 μm.

(d) AQP4 (left) and GFAP (right) relative immunofluorescence intensity in optic nerves across groups. n=6 eyes (3 sections per eye) for each group.

(e) C3d (green) and C5b-9 (red) staining in optic nerves across groups. Scale bars: 100 μm.

(f) C3d (left) and C5b-9 (right) relative immunofluorescence intensity in optic nerves across groups. n=6 eyes (3 sections per eye) for each group.

Data are shown as mean ± SEM, analysed via one-way ANOVA with Tukey's post hoc test. *$*p < 0.05$, $**p < 0.01$, $***p < 0.001$, $****p < 0.0001$*. Non-significant results omitted.

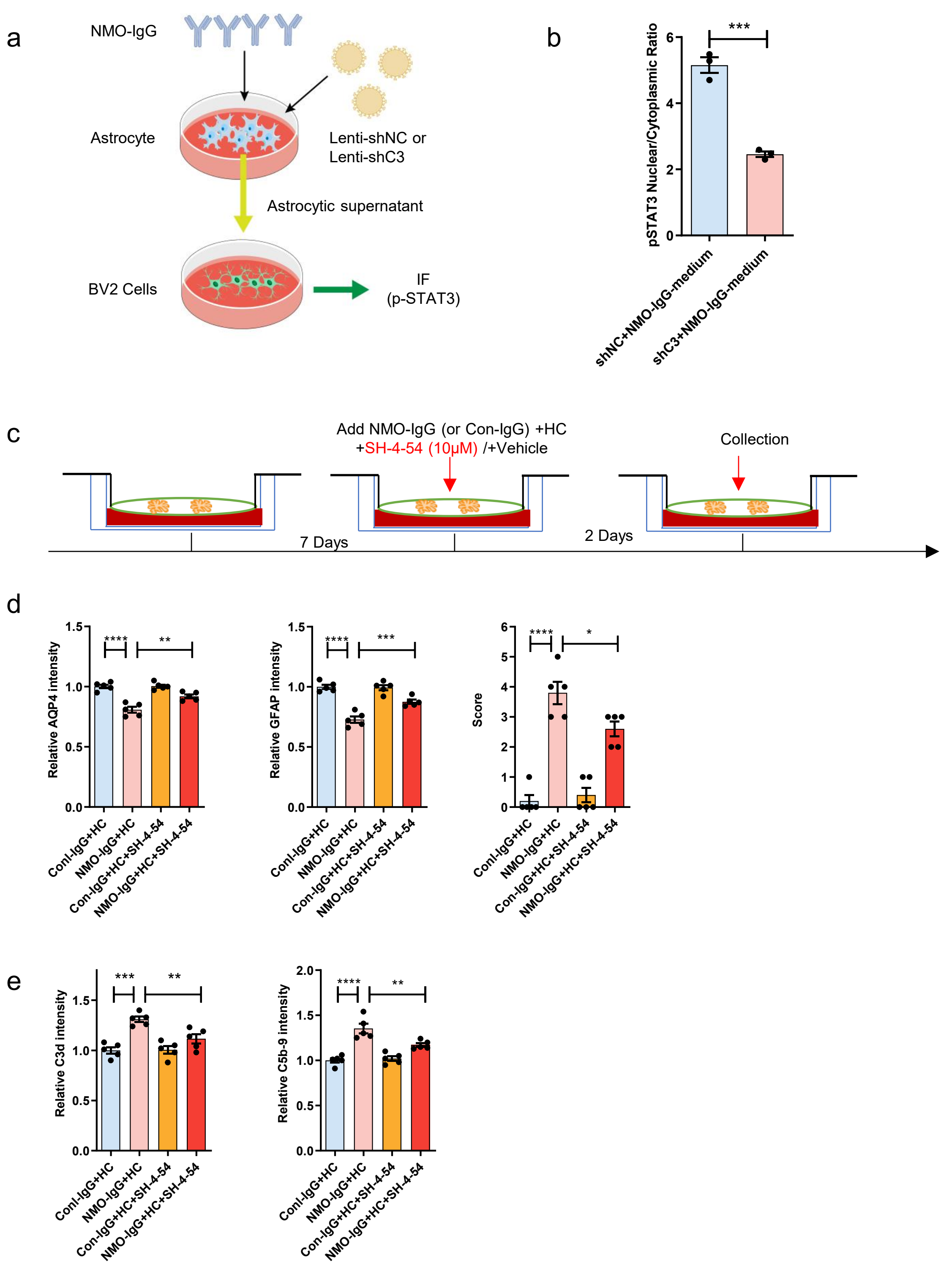

Figure S4. p-STAT3 inhibition, activated by C3-C3aR signaling, reduces astrocyte injury and complement activation in *ex vivo* NMO models

(a) Diagram of the in vitro cell assay setup.

(b) The nuclear/cytoplasmic ratio of p-STAT3 in BV2 cells after exposure to different astrocytic culture mediums for 24 hours. Scale: 20 μm. n=3 slides (6 sections per slide, 50 cells per section) for each group.

(c) Diagram illustrating the assay setup for the *ex vivo* NMO model.

(d) GFAP and AQP4 relative immunofluorescence intensity, alongside NMO-like lesion scoring, in cerebellar slices across groups. n=5 slices (3 sections per slice) for each group.

(e) C3d and C5b-9 relative immunofluorescence intensity in cerebellar slices across groups. n=5 slices (3 sections per slice) for each group.

Data are shown as mean ± SEM, analysed via Student's t test for (b) and one-way ANOVA with Tukey's post hoc test for other comparisons. *$^{*}p < 0.05$, $^{**}p < 0.01$, $^{***}p < 0.001$, $^{****}p < 0.0001$*. Non-significant results omitted.

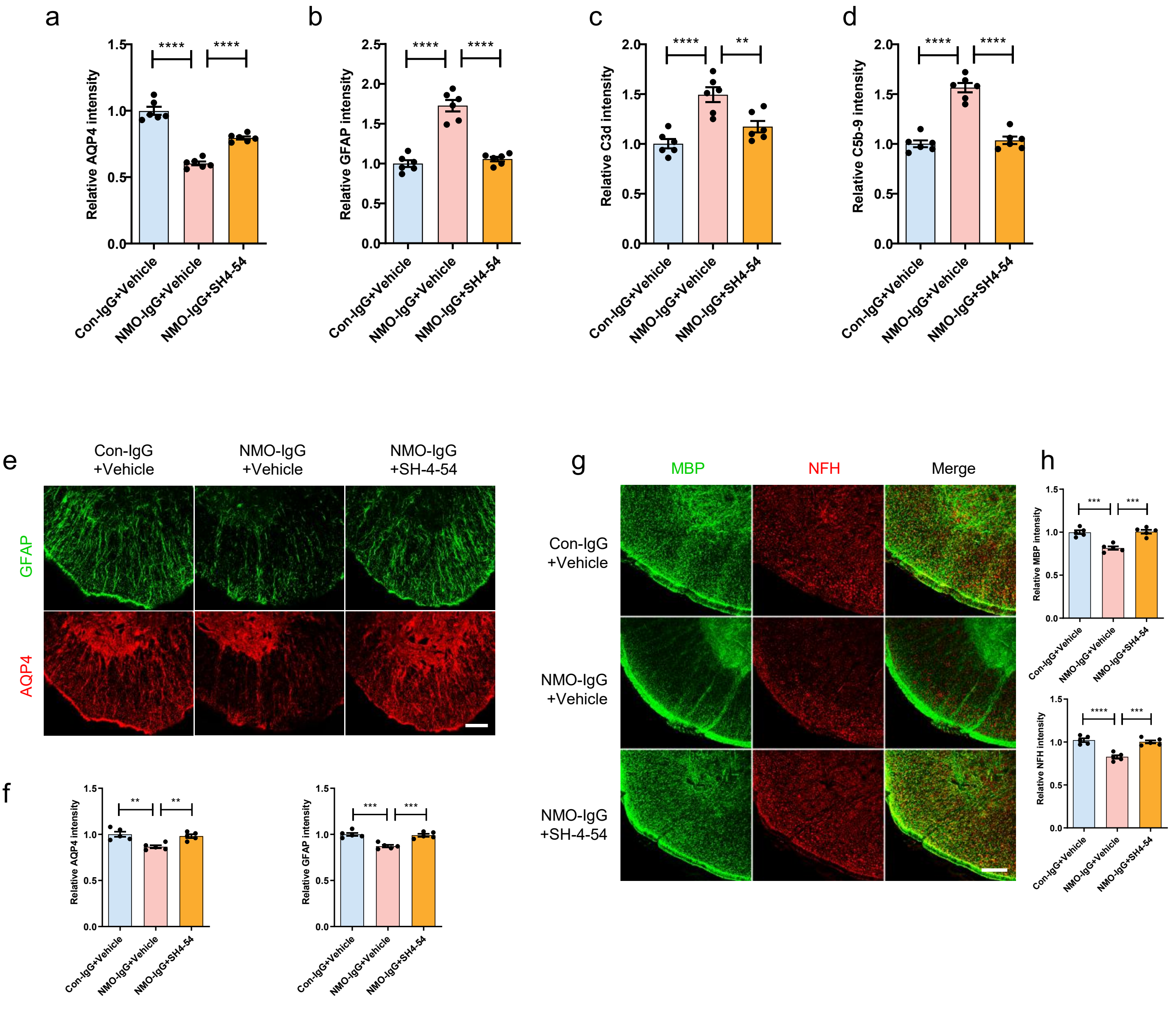

Figure S5. STAT3 inhibitor treatment mitigates NMO pathology *in vivo*

(a and b) AQP4 (a) and GFAP (b) relative immunofluorescence intensity in optic nerves across groups. n=6 eyes (3 sections per eye) for each group.

(c and d) MBP (c) and NFH (d) relative immunofluorescence intensity in optic nerves across groups. n=6 eyes (3 sections per eye) for each group.

(e) GFAP (green) and AQP4 (red) staining in spinal cord sections across groups. Scale: 100 μm.

(f) AQP4 and GFAP relative immunofluorescence intensity in spinal cord sections across groups. n=5 mice (3 sections per mouse) for each group.

(g) MBP (green) and NFH (red) co-staining in spinal cord sections across groups. Scale: 100 μm.

(h) MBP and NFH relative immunofluorescence intensity in spinal cord sections across groups. n=5 mice (3 sections per mouse) for each group.

Data are shown as mean ± SEM, analysed via one-way ANOVA with Tukey's post hoc test. *$p < 0.05$, **$p < 0.01$, ***$p < 0.001$, ****$p < 0.0001$. Non-significant results omitted.
