# Supplementary material for: Targeting C3aR/STAT3 signaling attenuates neuroinflammation and reverses neurological impairment in Neuromyelitis optica
Source: Fundam Res. 2025 Nov 7;6(5):3491–506. doi: 10.1016/j.fmre.2025.11.001 (PMC13644504; doi:10.1016/j.fmre.2025.11.001)

Uncropped gels

Fig.1B

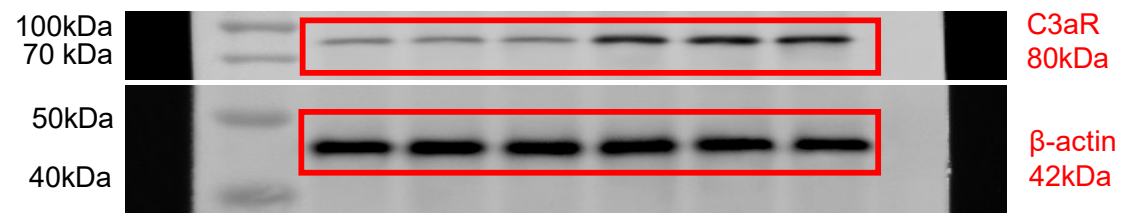

Fig.2B

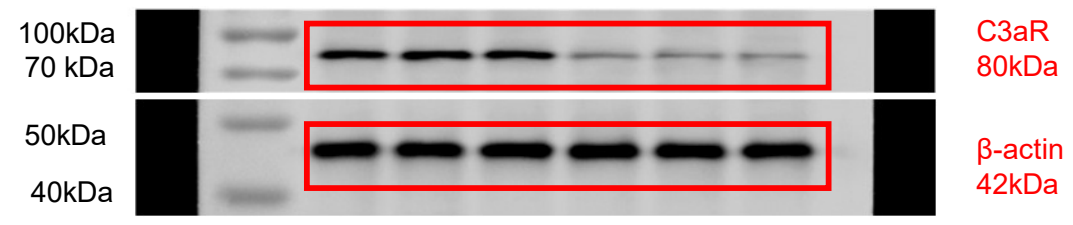

Fig.3O

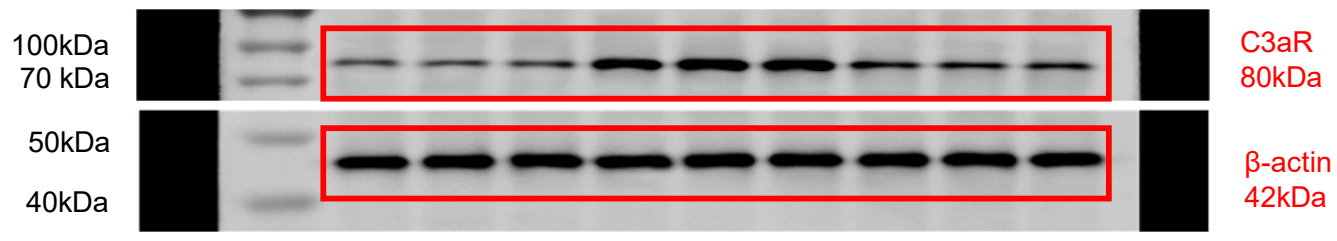

Fig.4G

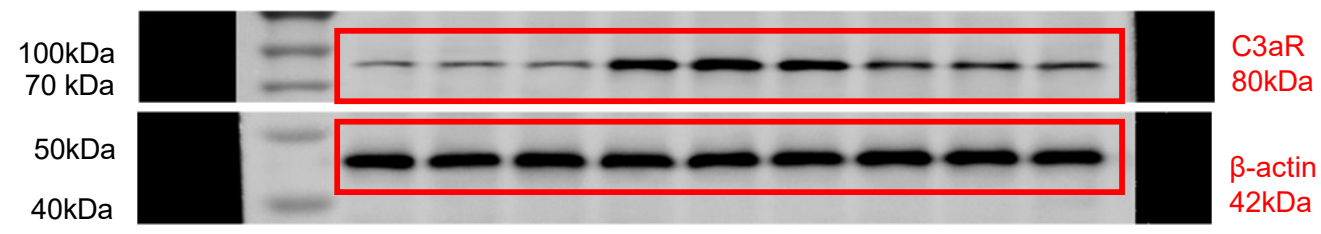

Fig.5C

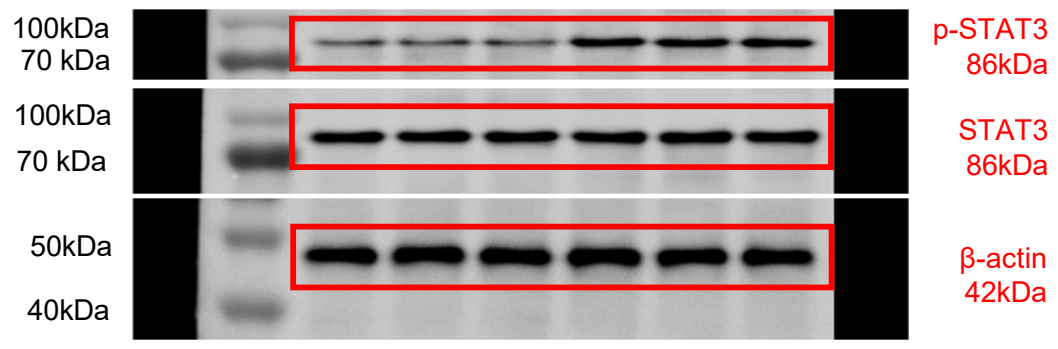

Fig.6Q

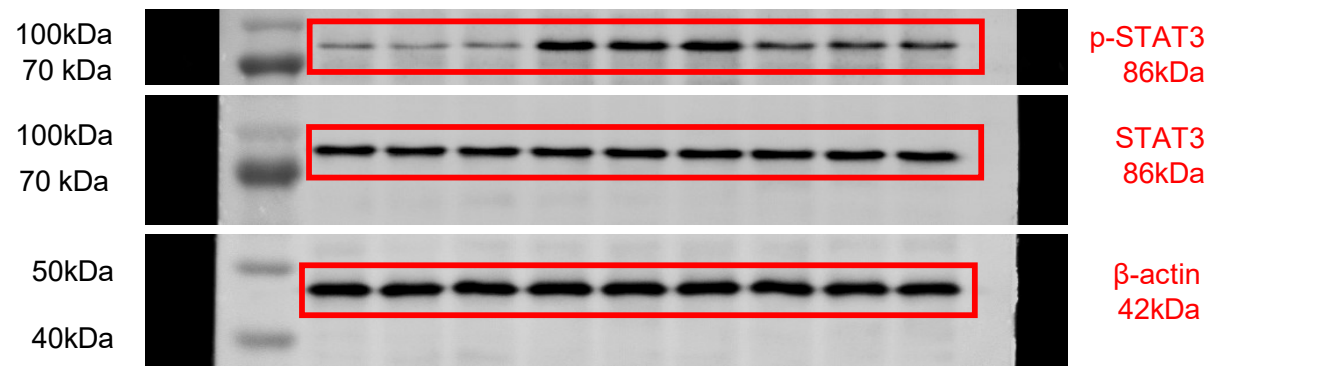

Fig.S1B

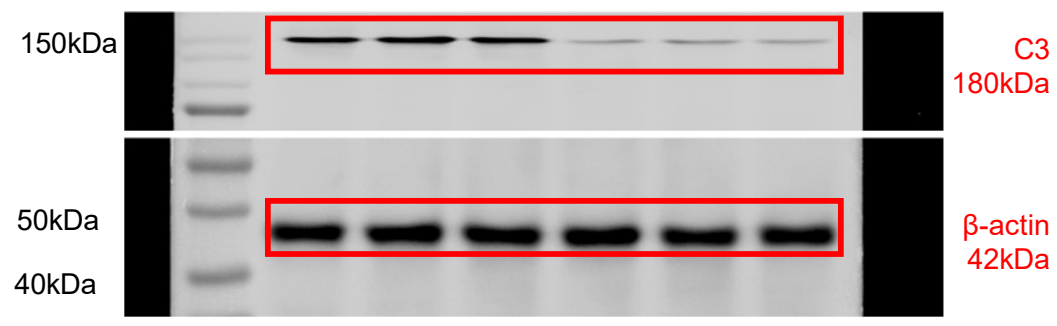

Supplement: Supplementary file 2 [file mmc2.pdf]
